# Genome-guided discovery and computational prioritization of next generation drug development from *Streptomyces* sp. VITGV156 (MCC 4965)

**DOI:** 10.3389/fmicb.2026.1736442

**Published:** 2026-03-30

**Authors:** Veilumuthu Pattapulavar, Manisha Shah, Praisy Joy Bell I, Sathiyabama Ramanujam, Saranyadevi Subburaj, Sivakumar Arumugam, Rajiniraja Muniyan, John Godwin Christopher

**Affiliations:** 1 Department of Biomedical Sciences, School of Biosciences and Technology, Vellore Institute of Technology, Vellore, India; 2 Department of Bio-Sciences, School of Biosciences and Technology, Vellore Institute of Technology, Vellore, India; 3 Department of Integrative Biology, School of Biosciences and Technology, Vellore Institute of Technology, Vellore, India; 4 Department of Science and Humanities, Karpagam Academy of Higher Education, Coimbatore, India; 5 Department of Biotechnology, Faculty of Engineering, Karpagam Academy of Higher Education, Coimbatore, India

**Keywords:** antibacterial metabolites, genome-guided prioritization, molecular docking, molecular dynamics simulation, secondary metabolites, *Streptomyces*, virtual screening

## Abstract

The rapid emergence of antimicrobial resistance necessitates the discovery of new bioactive metabolites and integrative strategies capable of accelerating antibiotic discovery. In this study, we employed a genome-guided experimental and computational workflow to investigate the antibacterial potential of *Streptomyces* sp. VITGV156 (MCC 4965). The ethyl acetate crude extract exhibited concentration-dependent antibacterial activity against Gram-positive and Gram-negative bacteria, including *Bacillus subtilis* (MTCC 2756), *Staphylococcus aureus* (MTCC 737), *Escherichia coli* (MTCC 1687), and *Klebsiella pneumoniae* (MTCC 109), with inhibition zones ranging from 24.33 ± 0.47 mm to 25.33 ± 0.47 mm at 100 μL, while the DMSO control showed no activity. Metabolomic profiling using GC–MS and LC–MS confirmed the production of diverse bioactive metabolites, providing experimental evidence supporting genome-mined biosynthetic potential. A total of 29 predicted secondary metabolites were subsequently prioritized using structure-based virtual screening against two clinically relevant antibiotic-resistance targets: the fluoroquinolone resistance protein QnrB1 and the tigecycline-inactivating monooxygenase Tet(X4). Docking validation confirmed the robustness of the computational protocol (RMSD = 1.71 Å), and several metabolites exhibited strong binding affinities (–5.0 to –12.3 kcal/mol). PASS bioactivity prediction and toxicity screening identified vicenistatin, prejadomycin, and ectoine as top candidates. Exhaustive docking and 200-ns molecular dynamics simulations further demonstrated stable protein–ligand interactions, particularly for vicenistatin, which enhanced structural stability of both target proteins. Overall, this study integrates antibacterial assays, metabolomics, genome mining, and molecular modeling to bridge the gap between genotype and phenotype, providing a rational pipeline for prioritizing natural products targeting antibiotic-resistance mechanisms. These findings highlight *Streptomyces* sp. VITGV156 as a promising source of antibacterial metabolites and support future purification and experimental validation of prioritized compounds.

## Introduction

The rapid global emergence of antimicrobial resistance (AMR) represents one of the most critical challenges to public health, significantly compromising the effectiveness of existing antibiotics and threatening the success of modern medical interventions. Pathogens resistant to frontline antibiotics, including fluoroquinolones and tetracyclines, are increasingly reported in both clinical and environmental settings, underscoring the urgent need for new antibacterial agents with novel mechanisms of action. In this context, natural products—particularly those derived from actinomycetes—continue to play a central role in antibiotic discovery, owing to their unparalleled chemical diversity and biological potency (Nazir et al., 2025).

Among actinomycetes, the genus *Streptomyces* has historically been the most prolific source of clinically important antibiotics, including tetracyclines, aminoglycosides, macrolides, and glycopeptides (De Simeis and Serra, 2021). Advances in whole-genome sequencing have revealed that *Streptomyces* genomes typically harbor far more biosynthetic gene clusters (BGCs) than the number of metabolites observed under standard laboratory conditions, indicating a substantial reservoir of cryptic or poorly expressed secondary metabolic potential (Meesil et al., 2025). Unlocking this hidden biosynthetic capacity remains a major challenge, as many BGCs are silent or expressed only under specific environmental or regulatory conditions (Pillay et al., 2022).

Genome-guided strategies have emerged as powerful tools for exploring the secondary metabolic potential of *Streptomyces* species. Bioinformatic platforms enable the identification, classification, and comparative analysis of BGCs, providing insights into the chemical families of metabolites encoded within microbial genomes (Lai et al., 2025).

Over the past decade, genome mining has become a central strategy for exploring the secondary metabolite biosynthetic capacity of *Streptomyces* species. Previous studies have successfully used antiSMASH-based analyses, comparative genomics, and metabolomics-guided prioritization to uncover novel biosynthetic gene clusters and cryptic metabolites from genome-sequenced strains (Leite et al., 2022). These approaches have revealed that a single *Streptomyces* genome may encode dozens of biosynthetic pathways, many of which remain uncharacterized or silent under laboratory conditions. Such studies highlight both the power and the limitations of genome mining, emphasizing the need for complementary experimental and metabolomic validation to connect biosynthetic potential with expressed chemical diversity.

Genome mining tools can reliably identify biosynthetic gene clusters and predict the chemical families of encoded metabolites; however, these predictions do not guarantee metabolite production under laboratory conditions (Leite et al., 2022). A single *Streptomyces* genome often contains numerous biosynthetic pathways, many of which remain transcriptionally silent or poorly expressed in standard culture media (Singh et al., 2022). Expression of these pathways is strongly influenced by environmental conditions, regulatory networks, and interspecies interactions. Consequently, genome-based predictions should be interpreted as indicators of biosynthetic potential rather than direct evidence of metabolite production (Lebedeva et al., 2021). Bridging this gap requires complementary experimental and metabolomic investigations to determine which biosynthetic pathways are active under specific cultivation conditions (Belknap et al., 2020; Lee et al., 2020).

When combined with computational approaches such as molecular docking, virtual screening, and molecular dynamics simulations, genome-based analyses can support the *prioritization* of candidate metabolites for further experimental investigation (Agu et al., 2023). However, it is important to recognize that such *in silico* predictions do not constitute experimental proof of biological activity and must be interpreted cautiously, particularly in the absence of direct metabolomic or genetic validation.

In addition to genome-based prediction, structure-based computational approaches are increasingly used to explore potential interactions between natural products and clinically relevant protein targets (Sadybekov and Katritch, 2023). Antibiotic resistance–associated proteins, including plasmid-mediated fluoroquinolone resistance proteins and antibiotic-modifying enzymes, represent attractive targets for evaluating the inhibitory potential of secondary metabolites (Ndagi et al., 2020). Docking-based screening can provide preliminary insights into binding affinity and interaction patterns, while molecular dynamics simulations offer a means to assess the stability of protein–ligand complexes in a dynamic environment (Noumi et al., 2025). Together, these approaches allow for hypothesis generation regarding potential modes of action, while acknowledging their inherent predictive limitations.

**FIGURE 1:**
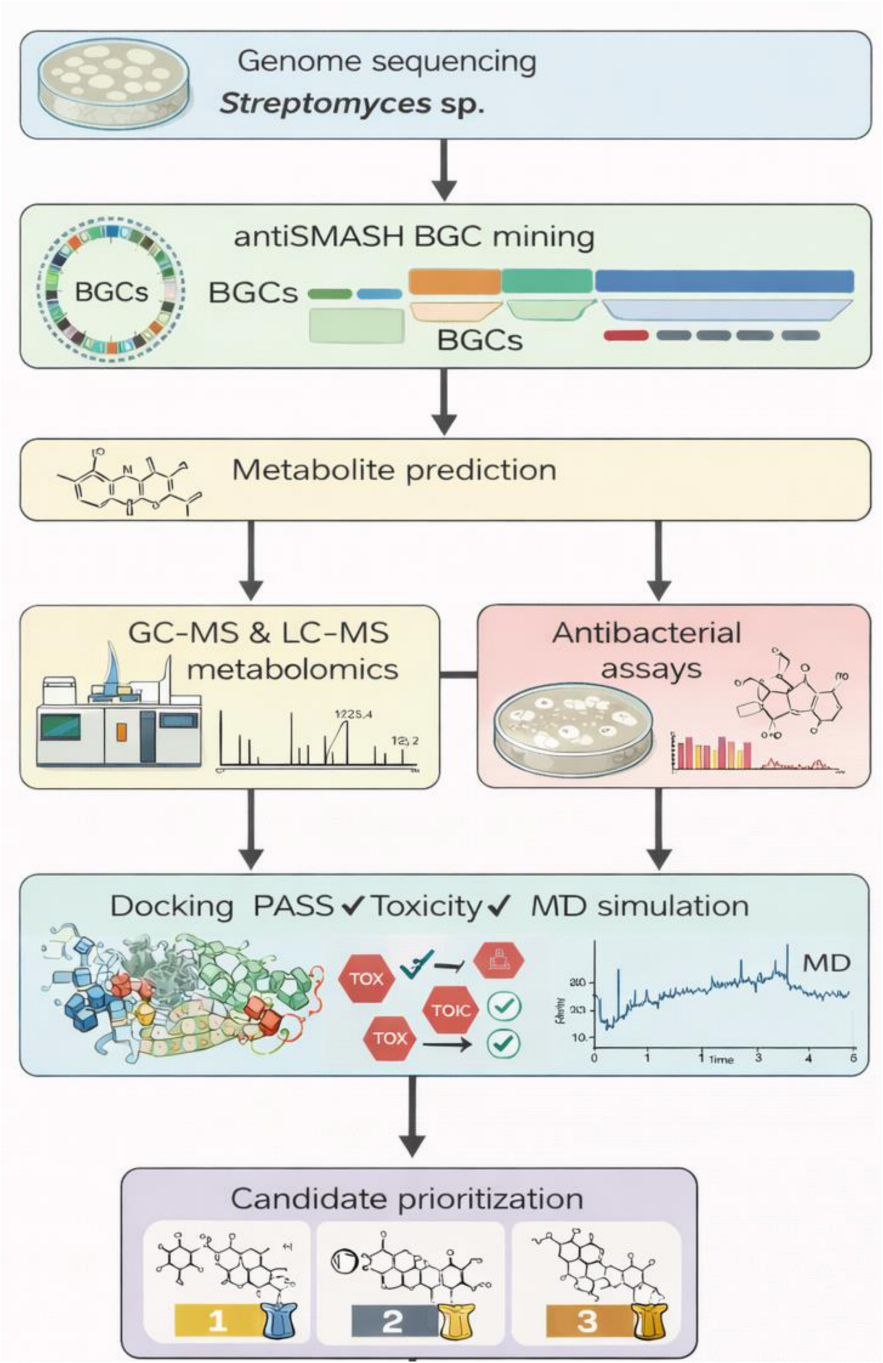
Integrated genome-guided discovery workflow used in this study. Schematic representation of the combined computational and experimental pipeline for antibacterial metabolite discovery. The workflow begins with whole-genome sequencing followed by biosynthetic gene cluster (BGC) mining and metabolite prediction. Experimental validation includes GC–MS and LC–MS metabolomic profiling and antibacterial activity screening. Predicted metabolites are further prioritized using molecular docking, PASS bioactivity prediction, toxicity assessment, and molecular dynamics simulations, leading to the selection of high-priority candidate antibacterial compounds for future validation.

*Streptomyces* sp. VITGV156 is a previously isolated and genomically characterized actinomycete that exhibits promising biosynthetic potential (Veilumuthu et al., 2024). Although its genome sequence has been reported, a systematic genome-guided evaluation of its secondary metabolite repertoire, coupled with computational prioritization against antibiotic resistance–associated targets, has not yet been comprehensively explored. Furthermore, limited experimental screening of crude extracts can provide preliminary phenotypic support for predicted antibacterial potential, while recognizing that such assays do not directly establish genotype–phenotype causality. Rather than introducing new bioinformatics software, the present study emphasizes the systematic integration of genome mining, metabolomics, antibacterial assays, and structure-based computational prioritization into a unified workflow designed to reduce the gap between genomic prediction and experimental evidence. A schematic overview of the integrated genome-guided discovery and Computational Prioritization of Next Generation Drug Development from *Streptomyces* sp. used in this study is presented in Figure 1.

In this study, we employed an integrated genome-guided and *in silico* workflow to characterize the antibacterial secondary metabolite potential of *Streptomyces* sp. VITGV156. Biosynthetic gene clusters were identified through genome annotation, and predicted secondary metabolites were computationally prioritized using structure-based docking, biological activity prediction, toxicity filtering, and molecular dynamics simulations against selected resistance-associated protein targets. To reduce the gap between genome mining predictions and biological evidence, the present study integrates computational analysis with antibacterial assays and metabolomic profiling (GC–MS and LC–MS). This multi-layer strategy provides experimental support for the expression of biosynthetic gene clusters and strengthens the connection between genomic potential and observed antibacterial activity. While this work does not claim definitive validation of individual biosynthetic pathways or purified compounds, it aims to provide a rational and cautious prioritization framework that can guide future targeted metabolomic, genetic, and biochemical investigations.

## Materials and methods

### *Streptomyces* sp. VITGV156

A pure culture of *Streptomyces* sp. VITGV156 (MCC 4965) was characterized through both phenotypic and genotypic analyses (Veilumuthu and Christopher, 2022). Morphological assessment using phase-contrast and scanning electron microscopy provided detailed insights into its structural attributes. Additionally, a previously isolated strain, *Streptomyces* sp. VITGV156, stored at -20°C in the Microbiology Lab at SBST, VIT University, was revived on ISP2 agar for further studies.

### Genome annotation and comparative genomic analysis

The whole genome of *Streptomyces* sp. VITGV156 was submitted to the NCBI SRA repository under Bio Project accession number PRJNA750872;^1^ BioSample accession number SAMN20499087;^2^ SRA accession number SRS9645416 and Accession ID: 20499087 and NCMR (Culture Deposit Accession no)—MCC4965. The datasets presented in this study can be found in online repositories. The names of the repository/repositories and accession number(s) can be found at: https://www.ncbi.nlm.nih.gov/biosample/20499087. The biosynthetic clusters involved in the synthesis of secondary metabolites were analyzed using antiSMASH 7.0 (Blin et al., 2023).

### Phylogenetic and phylogenomic analysis

The taxonomic position of strain VITGV156 was investigated using both 16S rRNA gene phylogeny and genome-based phylogenomic comparison, following current recommendations for *Streptomyces* systematics. The nearly complete 16S rRNA gene sequence was extracted from the assembled genome and compared with type strain sequences retrieved from the NCBI database. Multiple sequence alignment was performed using the ClustalW algorithm implemented in MEGA X. A phylogenetic tree was constructed using the Maximum Likelihood method with the Tamura–Nei model, and branch robustness was evaluated using 1,000 bootstrap replicates. To complement the 16S rRNA analysis, genome-scale relatedness was assessed using Average Nucleotide Identity (ANI) and digital DNA–DNA hybridization (dDDH) metrics derived from comparisons with closely related *Streptomyces* genomes identified through NCBI genome similarity searches. These metrics were interpreted using accepted species delineation thresholds (ANI ≈ 95–96%; dDDH ≈ 70%).

## Molecular docking and simulation studies

### Preparation of protein

The crystal structures of two antibiotic resistance-associated proteins, fluoroquinolone resistance protein QnrB1 and tigecycline-degrading monooxygenase Tet(X4), were selected for this study and retrieved from the Protein Data Bank^3^ in PDB format (Berman et al., 2000). Protein preparation involved removal of co-crystallized ligands and water molecules, correction of missing atoms, computation of partial charges (primarily Kollman charges), removal of unnecessary chains and heteroatoms, and addition of polar hydrogen atoms. The prepared protein structures were converted into PDBQT format for further docking analysis (Szklarczyk et al., 2023). The active sites of both proteins were identified using the CASTpFold server (Computed Atlas of Surface Topography of the Universe of Protein Folds) (Ye et al., 2024).

### Ligand preparation of secondary metabolites from *Streptomyces* sp. VITGV156

A total of 29 potential secondary metabolites identified from a biosynthetic gene cluster of *Streptomyces* sp. VITGV156 were selected for docking studies against the target proteins. These metabolites were retrieved from the PubChem database in SDF format. Canonical SMILES representations were generated using ChemSketch software. Geometry optimization and energy minimization were performed using the Open Babel minimization tool (O’Boyle et al., 2011). The compounds were converted from 2D to 3D structures using the MMFF94 force field via the Open Babel GUI, followed by geometry cleaning using ArgusLab (version 4.0.1). The optimized structures were saved in PDB format for docking studies.

### Self-docking validation

To validate the reliability of the docking protocol, self-docking was performed using the co-crystallized ligands bound to the target proteins. The grid box was defined to fully encompass the active site of each protein. Docking was carried out for 100 iterations, and the resulting ligand poses were analyzed and superimposed with the reference crystal structures to validate the docking accuracy (Sundararajan et al., 2023).

### Molecular interaction analysis and high-throughput virtual screening

High-throughput virtual screening was performed using AutoDock Vina (v1.1.2) (Trott and Olson, 2010). AutoDock Tools were used to convert the prepared proteins from PDB to PDBQT format (Morris et al., 2009). The docking configuration file (config.txt) was generated using Discovery Studio BIOVIA 2021. Docking of the secondary metabolites with both target proteins was automated using a Perl script (Gupta et al., 2022). Each metabolite was docked in 10 independent runs, and docking results were evaluated based on binding affinity scores. The optimal binding pose for each ligand was selected based on the lowest binding energy. The docked protein–ligand complexes were further analyzed using Discovery Studio BIOVIA 2021 to identify key molecular interactions.

### Identification of biological activity and toxicity analysis

The biological activities of the identified secondary metabolites were predicted using the PASS Online server. This tool predicts biological activity based solely on the chemical structure and provides two probabilistic parameters: Pa (probability of activity) and Pi (probability of inactivity), both ranging from 0 to 1. A higher Pa value indicates a greater likelihood of biological activity. In PASS predictions, antimicrobial activity scores range from 0 (inactive) to 1 (active). A *P*-value > 0.7 indicates strong activity, > 0.5 indicates moderate activity, and > 0.3 indicates low activity. This predictive framework enables prioritization of promising compounds for experimental validation (Filimonov et al., 2014). Compounds with favorable predicted biological activity were subjected to toxicity analysis using DataWarrior software. Toxic compounds, including those predicted to be mutagenic or tumorigenic, were excluded from further analysis. PASS distinguishes between general antibacterial activity and antibiotic-like activity. Antibacterial activity refers to broad bactericidal or bacteriostatic potential, whereas antibiotic activity represents prediction of mechanisms consistent with classical antibiotic modes of action.

### Exhaustive docking

The top three compounds that passed both biological activity prediction and toxicity screening were subjected to exhaustive docking using AutoDock Tools. Each ligand was docked for 100 iterations against both target proteins. Binding energies corresponding to the most populated clusters were selected for further analysis. Binding poses and protein-ligand interactions were visualized using Discovery Studio Visualizer (Karunakaran and Muniyan, 2023).

### Molecular dynamics simulation

Molecular dynamics simulations were performed using GROMACS 2024.3 to evaluate the dynamic stability of the protein-ligand complexes. The protein-ligand complexes obtained from exhaustive docking were used as initial structures. The CHARMM36 force field was applied to the proteins, while ligand topologies were generated using SwissParam server (Bell and Muniyan, 2025). Each complex was solvated in a triclinic box using the TIP3P water model, maintaining a minimum distance of 10 Å between the protein and box edges. Counter ions (Na^+^/Cl^–^) were added to neutralize the system. Energy minimization was carried out using the steepest descent algorithm until convergence was achieved. Following minimization, the systems were equilibrated under NVT (constant number of particles, volume, and temperature) for 100 ps at 300 K, followed by NPT (constant number of particles, pressure, and temperature) equilibration for 100 ps at 1 bar pressure using the Parrinello–Rahman barostat. Production MD simulations were performed for 200 ns with a time step of 2 fs. Long-range electrostatic interactions were treated using the Particle Mesh Ewald (PME) method, and all bond lengths were constrained using the LINCS algorithm. Trajectory analysis was conducted to calculate RMSD, RMSF, SASA, and radius of gyration (Rg) using standard GROMACS utilities. Hydrogen bond analysis was also performed to evaluate ligand stability within the binding pocket throughout the simulation (Bhavyashree et al., 2024).

### Metabolite production and extraction of bioactive compounds

To assess the biosynthetic potential of *Streptomyces* sp. VITGV156, the strain was cultivated in ISP2 broth supplemented sugarcane baggeese and incubated at 30°C for 15 days. For large-scale metabolite production, the strain was grown in 1,000 mL Erlenmeyer flasks containing 500 mL of ISP2 broth under identical conditions. Post-incubation, the culture broth was centrifuged at 10,000 rpm for 20 min to separate the supernatant from the biomass. Secondary metabolites were extracted using a two-phase solvent extraction method with ethyl acetate (1:1 ratio). The mixture was vigorously shaken for 10 min and incubated on a shaker at 200 rpm for 24 h. Following 30 min of phase separation, the organic layer was collected and concentrated using a rotary evaporator (model RE100-Pro) at 54°C and 80 rpm. The crude extract was then dried, weighed, dissolved in 200 μL of methanol, and stored at -20°C for subsequent analysis (Nisha et al., 2025). To experimentally support genome mining predictions, the crude ethyl acetate extract of *Streptomyces* sp. VITGV156 was analyzed using GC–MS and LC–MS. Both analyses revealed a chemically diverse metabolite profile consistent with the presence of multiple active biosynthetic pathways (Pattapulavar et al., 2025b).

### Secondary screening against bacterial strains

Antibacterial activity of the crude ethyl acetate extract of *Streptomyces* sp. VITGV156 was evaluated using the agar well diffusion method against four reference bacterial strains*: Bacillus subtilis* (MTCC 2756), *Staphylococcus aureus* (MTCC 737), *Escherichia coli* (MTCC 1687), and *Klebsiella pneumoniae* (MTCC 109). Mueller–Hinton agar plates were inoculated with freshly prepared bacterial suspensions (0.5 McFarland standard). Wells of 6 mm diameter were punched aseptically using a sterile cork borer. The crude extract was tested at four volumes (25, 50, 75, and 100 μL). Tetracycline served as the positive control, while dimethyl sulfoxide (DMSO) served as the negative control. Plates were incubated at 37°C for 24 h and the inhibition zones were measured in millimeters. Each experiment was performed in triplicate independent assays, and inhibition zones were measured in three perpendicular directions. Results are presented as mean ± standard deviation (SD) (Pattapulavar et al., 2025c).

### GC–MS analysis of the bioactive extract of VITGV156

Chemical profiling of the antibacterial ethyl acetate extract of *Streptomyces* sp. VITGV156 was performed using gas chromatography–mass spectrometry (GC–MS) to identify volatile and semi-volatile metabolites. Analyses were carried out on a Thermo Scientific Trace GC Ultra system coupled to an ISQ Quadrupole mass spectrometer equipped with a TG-5MS capillary column. Helium was used as the carrier gas at a constant flow rate of 1 mL min^–1^, and ionization was performed by electron ionization (EI) at 70 eV. The oven temperature program was: 50°C for 2 min, ramp to 150°C at 7°C min^–1^, ramp to 270°C at 5°C min^–1^ and ramp to 310°C at 3.5°C min^–1^. Mass spectra were acquired across a broad scan range to detect diverse metabolites. Compound identification was achieved by comparison with the NIST 14 Mass Spectral Library. Only peaks with a match factor ≥ 80% were accepted, and matches ≥ 90% were considered high-confidence identifications. Relative abundance of metabolites was estimated using peak area normalization, where each peak area was expressed as a percentage of the total ion chromatogram (TIC). Because external standards were not used, the analysis is considered semi-quantitative, consistent with standard microbial natural-product GC–MS workflows (Pattapulavar et al., 2025a).

### LC–MS analysis of the bioactive extract of VITGV156

To detect non-volatile and higher-molecular-weight metabolites associated with biosynthetic gene clusters, LC–MS analysis was performed. Chromatographic separation was achieved using a Phenomenex Kinetex C18 column (50 × 2.1 mm, 2.6 μm, 100 Å). High-resolution detection was carried out using a QTOF mass spectrometer equipped with an electrospray ionization (ESI) source operating in negative mode. Instrument parameters: Capillary voltage: 4.5 kV, Source temperature: 200°C and Scan rate: 1 Hz. Data were acquired across a wide m/z range to capture diverse metabolite classes. LC–MS/MS fragmentation spectra were collected for major precursor ions to assist compound-class annotation and comparison with genome-mined biosynthetic gene clusters (BGCs).

### Statistical analysis

Antibacterial activity data were analyzed using two-way analysis of variance (ANOVA) with treatment and volume as independent variables, followed by Tukey’s multiple comparison test using GraphPad Prism. A *p*-value < 0.05 was considered statistically significant.

## Results

### Phylogenetic and phylogenomic placement

**FIGURE 2:**
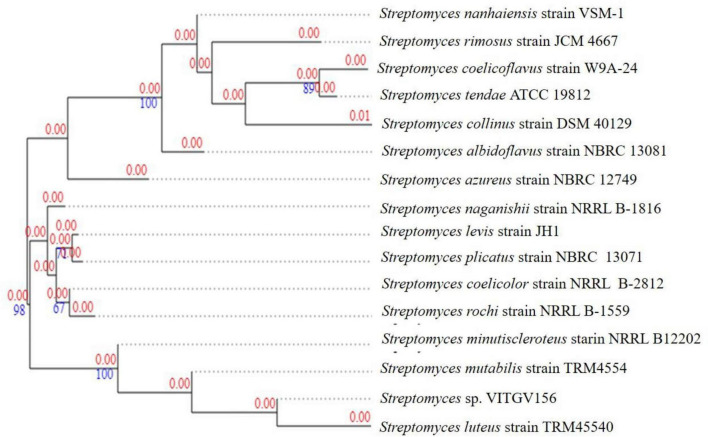
Genome-based phylogenetic tree of *Streptomyces* sp. VITGV156 generated using autoMLST. The tree was constructed based on concatenated alignment of 92 single-copy orthologous housekeeping genes identified automatically by the autoMLST pipeline. Phylogenetic reconstruction was performed using the Maximum Likelihood method with 1,000 bootstrap replicates. Bootstrap support values (%) are indicated at branch nodes. Red numbers represent branch lengths, and blue numbers indicate bootstrap support values. Only representative closely related species and selected distant taxa within the genus *Streptomyces* were included. SSU refers to small subunit ribosomal RNA.

The Maximum Likelihood phylogenetic tree based on 16S rRNA sequences placed strain VITGV156 firmly within the genus *Streptomyces*, forming a distinct lineage among closely related type strains (Supplementary Figure 1 and Figure 2). The strain clustered near members of the *Streptomyces griseicolor* clade but formed an independent branch supported by strong bootstrap values, indicating clear evolutionary divergence. Genome-based comparison further supported this observation. ANI and dDDH values between VITGV156 and its closest reference genomes were below the accepted species delineation thresholds, suggesting that the strain represents a distinct genomic lineage within the genus. Together, these results indicate that strain VITGV156 likely represents a putative novel *Streptomyces* species candidate. Formal taxonomic description will be performed in a dedicated polyphasic taxonomic study.

**FIGURE 3:**
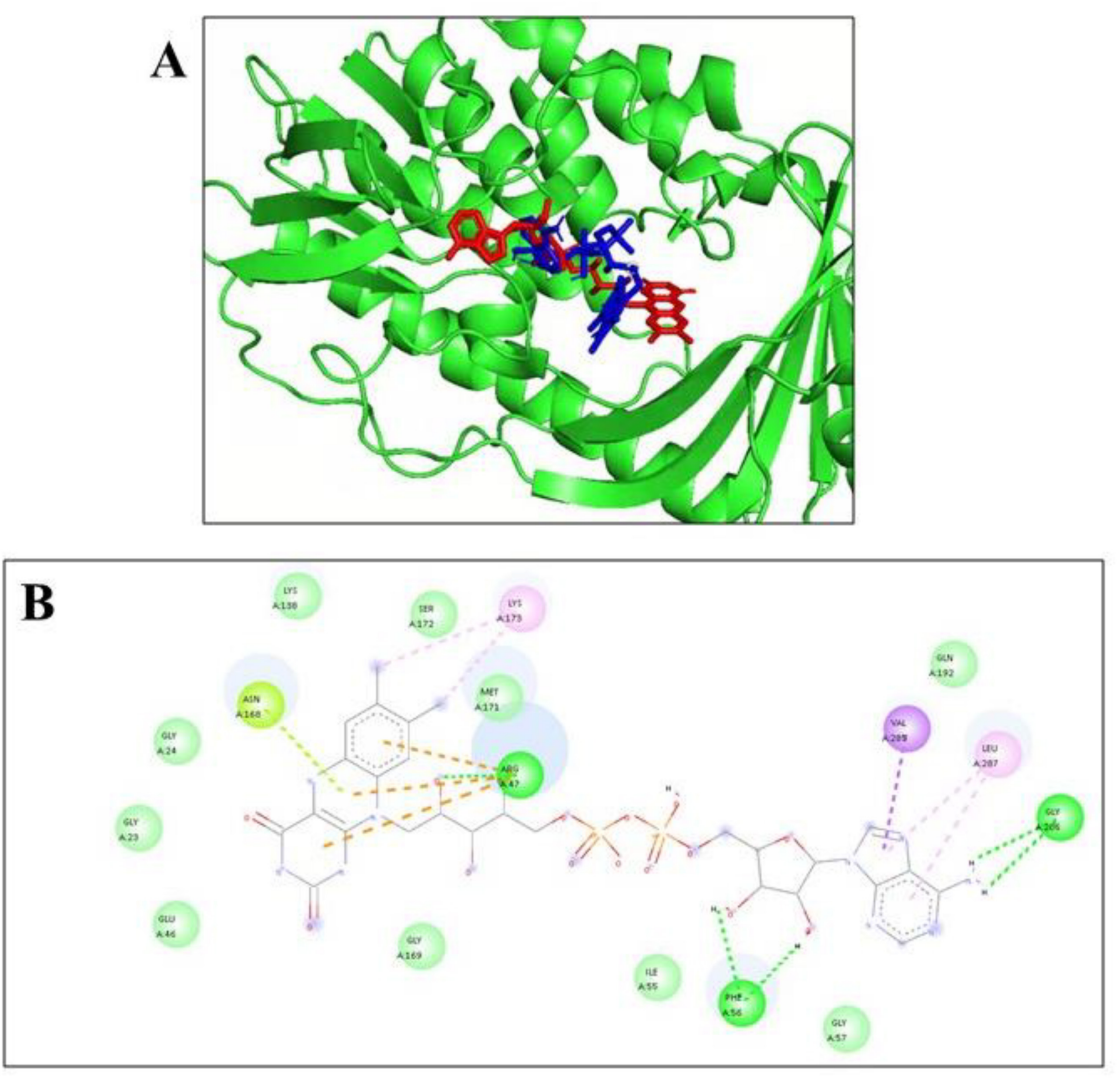
**(A)** 3D representation of the superimposed ligands **(B)** 2D interaction of the self-docked complex.

The 16S rRNA gene sequence of strain VITGV156 was aligned with closely related *Streptomyces* type strains retrieved from the NCBI database. Multiple sequence alignment was performed using MUSCLE, and a phylogenetic tree was constructed using the Maximum Likelihood method with 1,000 bootstrap replicates in MEGA X. For genome-based phylogenetic analysis, autoMLST was employed to identify conserved single-copy orthologous housekeeping genes across representative *Streptomyces* genomes. A concatenated alignment of 92 orthologous genes was generated, and a Maximum Likelihood tree was inferred using default parameters with 1,000 bootstrap replicates. Only closely related species identified in the 16S analysis and representative distant taxa were included to ensure consistency between analyses. To ensure robust taxonomic placement, both 16S rRNA gene-based and genome-based phylogenetic analyses were performed using overlapping representative taxa. In the 16S rRNA tree (Figure 2), strain VITGV156 clustered closely with *Streptomyces luteus* TRM45540 and *Streptomyces mutabilis* TRM4554, supported by high bootstrap values. Similarly, in the genome-based multi-locus species tree (Figure 3), VITGV156 grouped within the same clade containing these closely related species, demonstrating congruent taxonomic placement. Minor differences in branching order were observed between the two trees, which can be attributed to the higher resolution of genome-scale data compared to single-gene analysis. However, both phylogenetic approaches consistently placed strain VITGV156 within the genus *Streptomyces* and supported its close relationship with the same neighboring taxa. These results confirm the stable taxonomic positioning of strain VITGV156 and demonstrate concordance between single-gene and genome-wide phylogenetic inference.

### Protein and ligand preparation

The crystal structure of fluoroquinolone resistance protein QnrB1 (PDB ID: 2XTY), comprising 217 amino acid residues with a resolution of 1.80 Å, and tigecycline-degrading monooxygenase Tet(X4) (PDB ID: 7EPV), comprising 388 amino acid residues with a resolution of 1.78 Å were selected for this study (Cheng et al., 2021). Missing loop regions in 7EPV (residues 4–11, 247–249, and 384–388) were modeled using the Modeler plugin in UCSF Chimera. No mutations were observed in either protein structure. Grid boxes were defined to encompass the active site residues. For 7EPV, grid dimensions were set to 126 × 126 × 126 Å with center coordinates at (*x* = 5.47, *y* = –30.53, *z* = –4.52), while for 2XTY, the grid dimensions were identical with center coordinates at (*x* = –30.82, *y* = 10.10, *z* = –19.21). All 29 metabolites were energy-minimized using the MMFF94 force field prior to docking.

### Docking validation study

The co-crystallized ligand of Tet(X4) (PDB ID: 7EPV) was identified as flavin adenine dinucleotide (FAD). Redocking of FAD yielded a binding energy of –13.23 kcaL/moL. Hydrogen bond formation is observed between three key residues Arg47, Phe 56 and Gly 286. Superimposition of the redocked and crystal ligand conformations resulted in an RMSD value of 1.71 Å, as shown in the Figure 3. This confirms that the docking protocol is reliable and accurate.

### Virtual screening of secondary metabolites

All 29 secondary metabolites were docked against the two antibiotic resistant proteins. AutoDock Vina generated nine binding conformations for each ligand, from which the best-scoring pose was selected. The docking results revealed that the majority of the screened metabolites exhibited favorable binding affinities toward both target proteins. Binding energies ranged from –5.0 to –12.2 kcaL/moL, indicating moderate to strong binding interactions (Supplementary Table 1). Several metabolites demonstrated binding energies comparable to or better than known inhibitors, highlighting their potential as effective modulators of antibiotic resistance proteins.

### Biological activity prediction and toxicity analysis

PASS analysis predicted antimicrobial, antifungal, and antibiotic activities for the metabolites, with Pa values ranging from 0.107 to 0.878 (Supplementary Table S2). Notably, prejadomycin, ectoine, and vicenistatin exhibited strong predicted antimicrobial activity. These compounds showed low structural similarity to known antibiotics, reducing the likelihood of cross-resistance. Following toxicity analysis, prejadomycin, ectoine, and vicenistatin were identified as non-mutagenic, non-tumorigenic, and non-irritant, and were selected for further studies (Table 1).

**TABLE 1:** Toxicity assessment of selected candidate antibacterial compounds against target proteins.

Metabolite	Mutagenic	Tumorigenic	Reproductive effective	Irritant	H-acceptors	H-donors	Total surface area	Druglikeness
Prejadomycin	None	None	None	None	5	3	222.81	0.009794
Ectoine	None	None	None	None	4	2	110.81	0.3535
Vicenistatin	None	None	None	None	6	3	426.06	0.90583

*In silico* toxicity risk assessment and drug-likeness properties of top-ranked antibacterial candidate compounds selected based on docking and PASS analysis. Toxicity parameters were predicted using OSIRIS DataWarrior. Risk categories are reported as none, low, or high risk. Drug-likeness, hydrogen bond donors, hydrogen bond acceptors, and total surface area were calculated to evaluate pharmacokinetic properties.

### Exhaustive docking

Following high-throughput virtual screening and toxicity filtering, the top three secondary metabolites prejadomycin, ectoine, and vicenistatin were subjected to exhaustive docking analysis to refine binding accuracy and evaluate pose stability. Exhaustive docking was performed using AutoDock Tools with 100 independent docking runs for each ligand against both Tet(X4) and QnrB1 proteins, employing the same grid parameters used during virtual screening.

**FIGURE 4:**
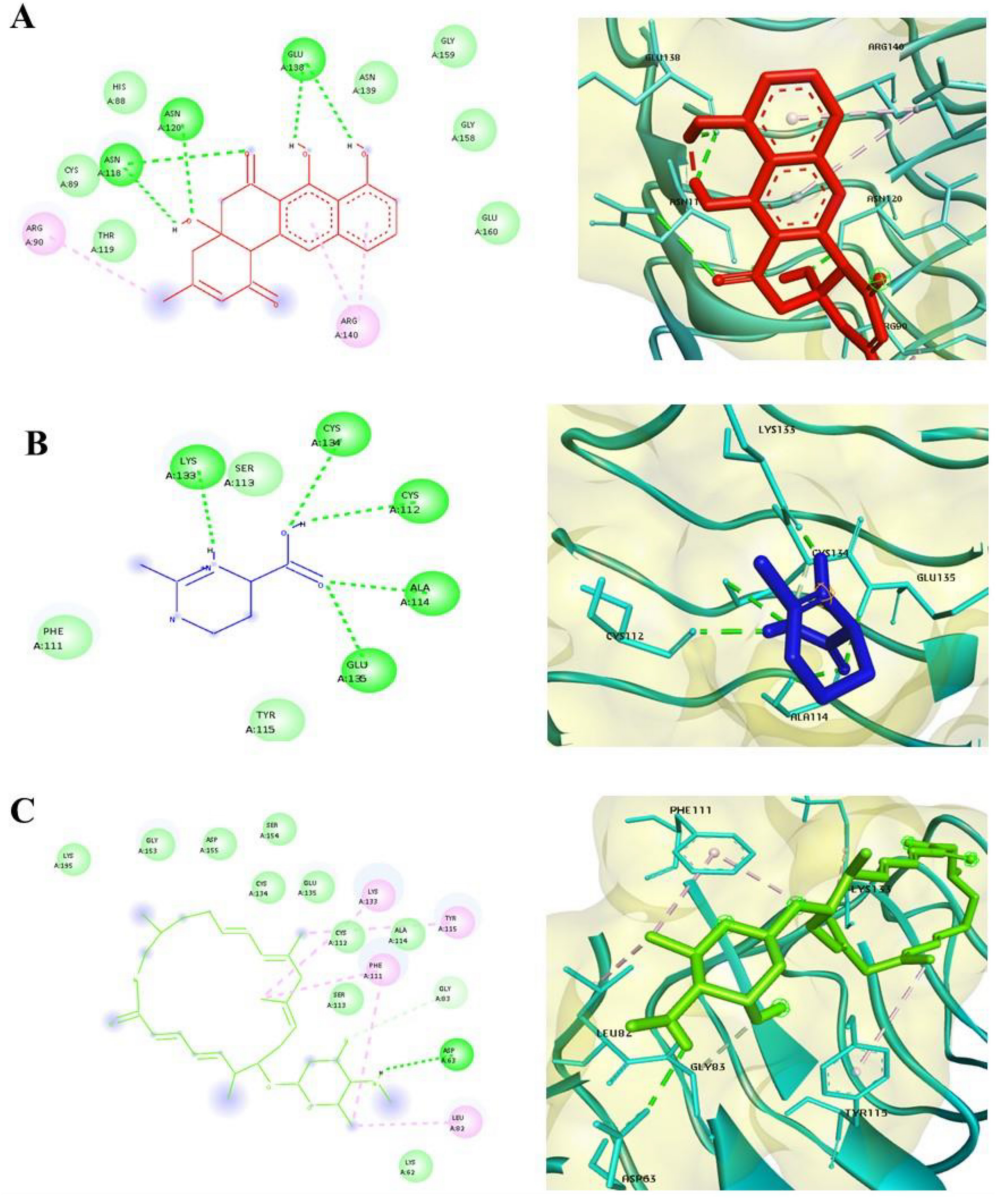
2D and 3D interaction of 2XTY and **(A)**. Prejadomycin **(B)**. Ectoine **(C)**. Vicenistatin.

**FIGURE 5:**
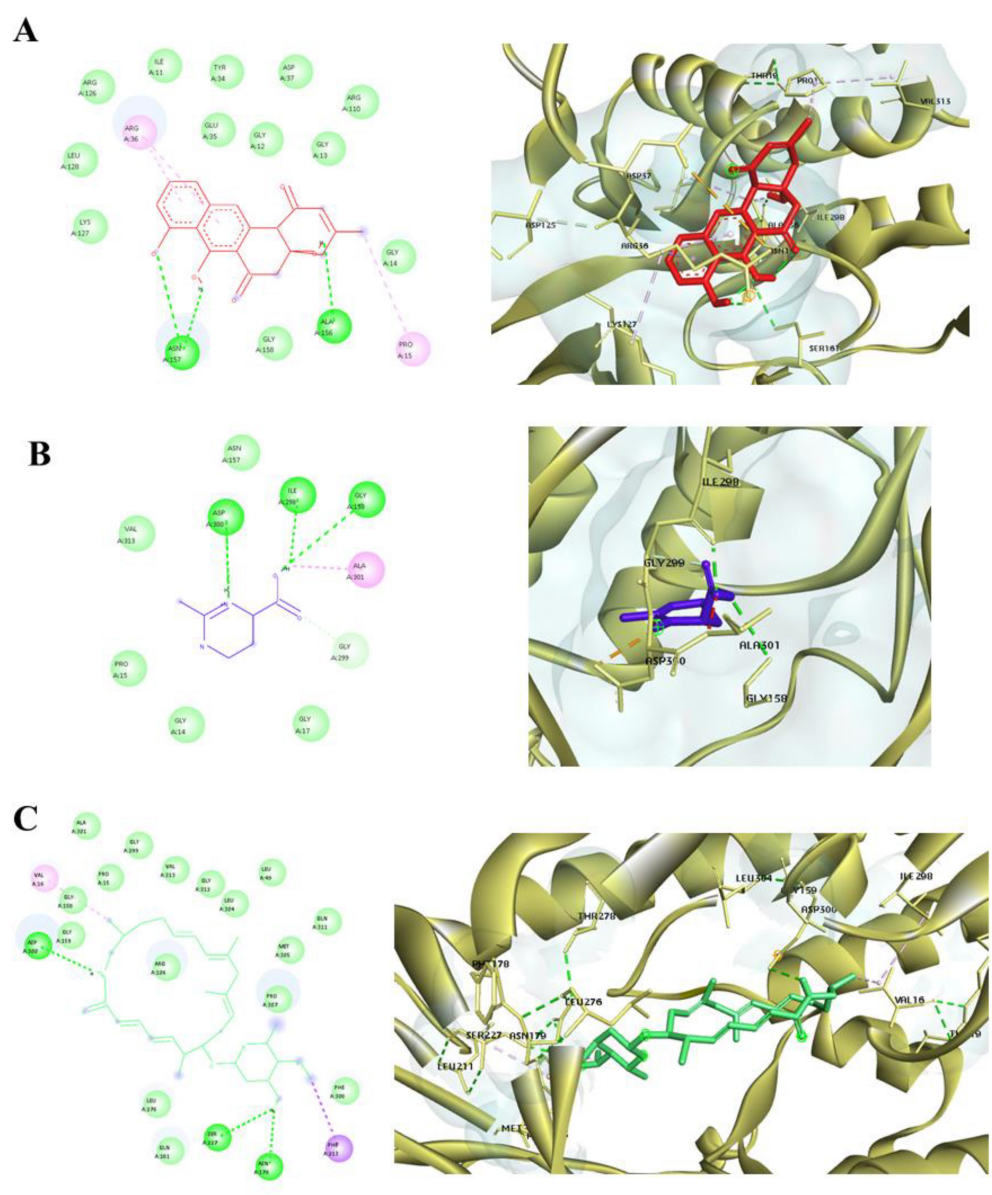
2D and 3D interaction of 7EPV and **(A)**. Prejadomycin **(B)**. Ectoine **(C)**. Vicenistatin.

The exhaustive docking results revealed consistently strong binding affinities for all three ligands toward both target proteins. The lowest binding energies ranged between -9.8 and -13.6 kcaL/moL, confirming high binding potency and stable ligand accommodation within the active site. Notably, the majority of the generated docking conformations clustered into dominant clusters, indicating convergence toward energetically favorable binding modes and high pose reproducibility. Vicenistatin exhibited the most favorable binding energy and the highest cluster population for both Tet(X4) and QnrB1, suggesting strong and stable binding preferences. Prejadomycin also demonstrated robust binding with well-defined clustering, whereas ectoine showed slightly higher binding energies but maintained consistent binding orientations across multiple runs. Detailed interaction analysis of the best-scoring poses was performed using Discovery Studio Visualizer (Figures 4, 5). In the Tet(X4)–ligand complexes, all three metabolites were observed to occupy the catalytic pocket involved in antibiotic degradation. Key interactions included hydrogen bonds with conserved residues, π–π stacking with aromatic amino acids, and hydrophobic contacts that stabilized ligand positioning within the binding cavity. For the QnrB1 protein, the selected ligands bound within the quinolone-interaction groove, forming stable hydrogen bonds and van der Waals interactions with residues known to mediate DNA protection. Vicenistatin formed multiple hydrogen bonds and hydrophobic interactions, contributing to its superior binding affinity and consistent docking orientation.

### Molecular dynamic simulation

**FIGURE 6:**
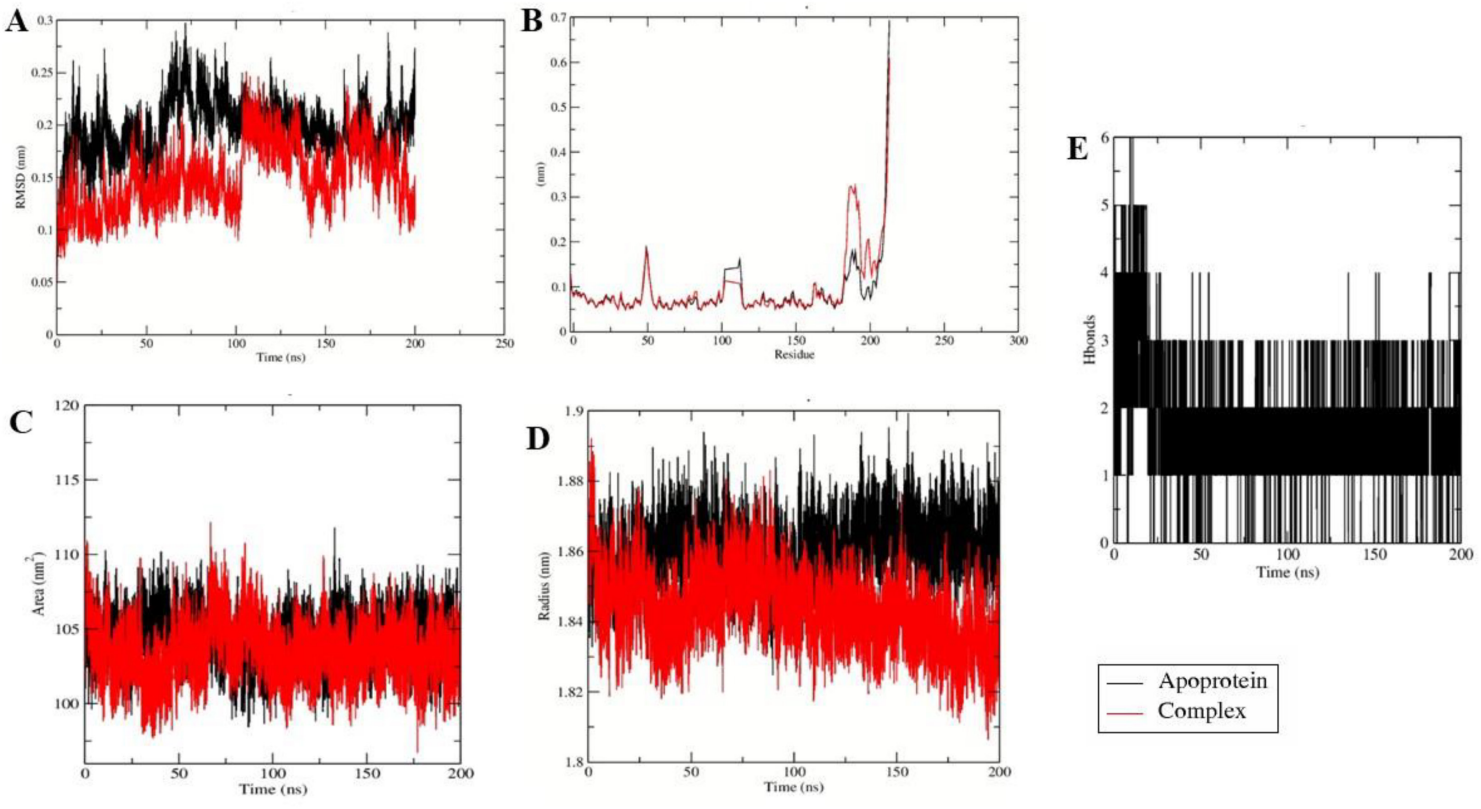
Molecular dynamic simulation results of 2XTY. **(A)** RMSD **(B)** RMSF **(C)** SASA **(D)** Rg **(E)** Hydrogen bonds.

**FIGURE 7:**
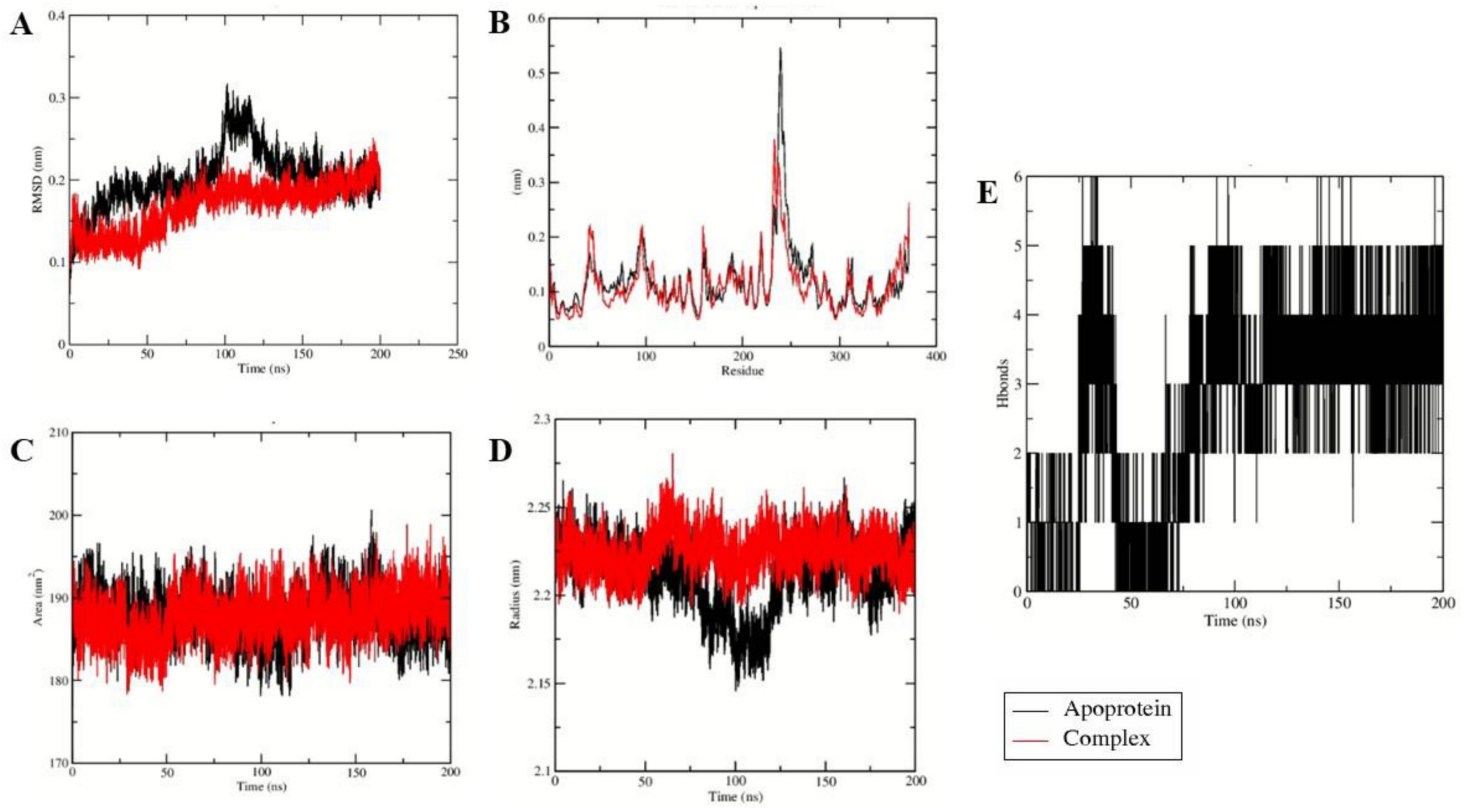
Molecular dynamic simulation results of 7EPV. **(A)** RMSD **(B)** RMSF **(C)** SASA **(D)** Rg **(E)** Hydrogen bonds.

To investigate the effect of ligand binding on protein stability and dynamics, 200 ns molecular dynamics simulations were performed for the apo forms of Tet(X4) and QnrB1, as well as their vicenistatin-bound complexes. Comparative analyses were carried out using RMSD, RMSF, radius of gyration (Rg), and solvent-accessible surface area (SASA) to elucidate ligand-induced conformational changes (Figures 6, 7).

In the case of fluoroquinolone resistance protein QnrB1 (PDB ID: 2XTY), RMSD analysis (Figure 7A) shows that the vicenistatin complex attains equilibrium early and remains stable with a lower average backbone deviation (0.14 nm) compared to the apoprotein (0.19 nm), indicating enhanced global stability upon ligand binding. Consistently, RMSF analysis (Figure 7B) demonstrates reduced residue-level flexibility in the ligand-bound system (0.086 nm) relative to the apoprotein (0.094 nm), particularly in loop and binding-site regions, suggesting ligand-induced stabilization of flexible residues. The SASA profile (Figure 7C) reveals a slightly lower solvent-exposed surface area for the complex (103.5 nm^2^) compared to the apoprotein (104.3 nm^2^), implying partial burial of surface residues upon vicenistatin binding. Furthermore, the radius of gyration (Figure 7D) indicates that the complex maintains a more compact and stable fold (1.85 nm) than the apoprotein (1.87 nm), which shows greater structural fluctuations.

Importantly, hydrogen bond analysis (Figure 7E) confirms the formation of persistent intermolecular hydrogen bonds between 2XTY and vicenistatin, with an average of 6 hydrogen bonds maintained throughout the simulation, supporting strong and stable protein-ligand interactions.

**FIGURE 8:**
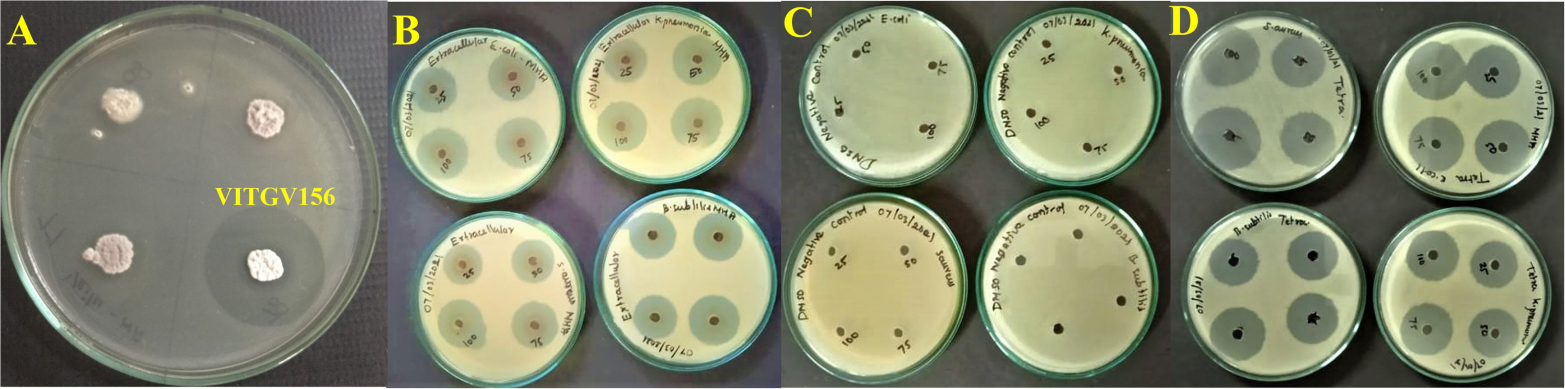
Antibacterial activity of *Streptomyces* sp. VITGV156. **(A)** Primary screening showing antagonistic activity against *Escherichia coli* (MTCC 1687) using the dual culture assay. **(B)** Antibacterial activity of the crude extract against Gram-negative bacteria [*Escherichia coli* (MTCC 1687) and *Klebsiella pneumoniae* (MTCC 109)] and Gram-positive bacteria [*Bacillus subtilis* (MTCC 2756) and *Staphylococcus aureus* (MTCC 737)] using the agar well diffusion method. **(C)** Negative control (DMSO) showing no inhibition zones. **(D)** Comparative inhibition zones produced by tetracycline (positive control) and the crude extract against selected pathogens.

With respect to tigecycline-degrading monooxygenase Tet(X4) (PDB ID: 7EPV), a similar pattern was observed. As shown in the Figure 8A, the backbone RMSD of the ligand bound complex remained stable and consistent with an average value of 0.16 nm than the apoprotein (0.20 nm) denoting efficient global stability upon binding of ligand. The RMSF profile (Figure 8B) further revealed reduced residue-level fluctuations in the complex (average RMSF: 0.11 nm) relative to the apoprotein (0.25 nm), particularly across non-terminal and flexible loop regions, suggesting ligand-induced stabilization of dynamic residues. Analysis of solvent-accessible surface area (SASA) (Figure 8C) showed slightly lower values for the complex (186.44 nm^2^) compared to the apoprotein (187.91 nm^2^), implying partial burial of surface residues and formation of a stable protein–ligand interface. Consistently, the radius of gyration (Figure 8D) demonstrated that the ligand-bound complex maintained a more compact conformation (2.12 nm) than the apoprotein (2.98 nm), indicating reduced structural dispersion upon ligand binding. Furthermore, hydrogen bond analysis (Figure 8E) revealed persistent intermolecular hydrogen bonding throughout the simulation, with an average of 2.1 ± 0.8 hydrogen bonds, highlighting stable interactions that contribute to ligand retention within the binding pocket. Overall, the quantitative MD results demonstrate that vicenistatin binding significantly enhances the stability, compactness, and conformational integrity of both 2XTY and 7EPV proteins. However, further experimental investigation needs to be done to assess the *in vitro* activity of vicenistatin.

### Antimicrobial activity of *Streptomyces* sp. VITGV56 crude extract

The crude ethyl acetate extract of *Streptomyces* sp. VITGV156 demonstrated dose-dependent antibacterial activity against both Gram-positive and Gram-negative bacteria (Figure 8 and Table 2). No inhibition zones were observed for the DMSO negative control, confirming that the antibacterial activity was attributable to the extracted metabolites. At the highest tested volume (100 μL), the extract produced inhibition zones ranging from 24.33 ± 0.47 mm to 25.33 ± 0.47 mm, indicating broad-spectrum antibacterial potential. Among the tested organisms, *Klebsiella pneumoniae* showed the highest susceptibility, followed by *Bacillus subtilis* (MTCC 2756), *Staphylococcus aureus* (MTCC 737), *Escherichia coli* (MTCC 1687), and *Klebsiella pneumoniae* (MTCC 109). A clear concentration-dependent increase in inhibition zone diameter was observed across all test organisms. At 25 μL, inhibition zones ranged between 20.33 ± 1.24 mm and 22.33 ± 0.47 mm, which increased to 24.33 ± 0.47 mm to 25.33 ± 0.47 mm at 100 μL. As expected, tetracycline exhibited stronger antibacterial activity than the crude extract. However, the extract showed notable inhibitory activity, achieving approximately 70–75% of the inhibition zone produced by tetracycline at comparable concentrations, indicating promising antibacterial potential of VITGV156 metabolites. Gram-negative bacteria [*Escherichia coli* (MTCC 1687), and *Klebsiella pneumoniae* (MTCC 109)] showed inhibition zones comparable to Gram-positive strains, suggesting that the extract contains metabolites with broad-spectrum antibacterial properties.

**TABLE 2:** Antibacterial activity of *Streptomyces* sp. VITGV156 crude extract against selected human pathogens measured by agar well diffusion assay.

Treatment	Volume (μ L)	*B. subtilis* (MTCC 2756)	*S. aureus* (MTCC 737)	*K. pneumoniae* (MTCC 109)	*E. coli* (MTCC 1687)
VITGV156 extract	25	20.33 ± 1.24	20.67 ± 0.47	22.33 ± 0.47	20.33 ± 0.47
	50	21.67 ± 1.24	21.67 ± 0.47	23.33 ± 0.47	21.67 ± 0.47
	75	22.67 ± 0.47	23.33 ± 0.47	24.33 ± 0.47	23.67 ± 0.47
	100	24.33 ± 0.47	24.33 ± 0.47	25.33 ± 0.47	24.67 ± 0.47
Tetracycline	25	34.00 ± 0.82	26.33 ± 0.94	34.00 ± 0.82	24.67 ± 3.29
	50	34.00 ± 0.82	27.67 ± 0.47	34.00 ± 0.82	26.67 ± 1.89
	75	36.33 ± 0.47	29.33 ± 0.47	36.33 ± 0.47	27.67 ± 1.89
	100	35.00 ± 1.63	30.33 ± 0.47	35.00 ± 1.63	29.00 ± 1.41
DMSO	25–100	0	0	0	0

Values represent inhibition zone diameter (mm), expressed as mean ± SD (*n* = 3). Tetracycline was used as positive control and DMSO as negative control. Statistical analysis was performed using two-way ANOVA (Supplementary Table S1).

All antibacterial assays were performed using three independent biological replicates. For each replicate, *Streptomyces* sp. VITGV156 was cultured in a separate fermentation batch and crude extract was independently prepared. Inhibition zones were measured in three perpendicular directions for each plate and averaged to minimize measurement bias.

### Statistical validation of antibacterial activity

To rigorously evaluate differences between the crude extract and tetracycline, inhibition zone data were subjected to two-way ANOVA followed by Tukey’s multiple comparison test (Supplementary Table 3 and Supplementary Figure 2). Statistical analysis revealed that both treatment type and extract volume significantly influenced antibacterial activity across all tested organisms (*p* < 0.001). For *Bacillus subtilis*, *Staphylococcus aureus*, and *Klebsiella pneumoniae*, significant interaction effects between treatment and volume were observed (*p* ≤ 0.0027), indicating dose-dependent differences between tetracycline and the crude extract. For *Escherichia coli*, the interaction effect was not significant (*p* = 0.0907), although both treatment and volume independently showed highly significant effects (*p* < 0.001). Overall, tetracycline produced significantly larger inhibition zones than the crude extract across all concentrations (*p* < 0.05). Nevertheless, the extract demonstrated substantial antibacterial activity, achieving approximately 70–75% of tetracycline efficacy, confirming the statistically significant yet promising antibacterial potential of *Streptomyces* sp. VITGV156 metabolites.

### GC–MS profiling of the antibacterial crude extract

**FIGURE 9:**
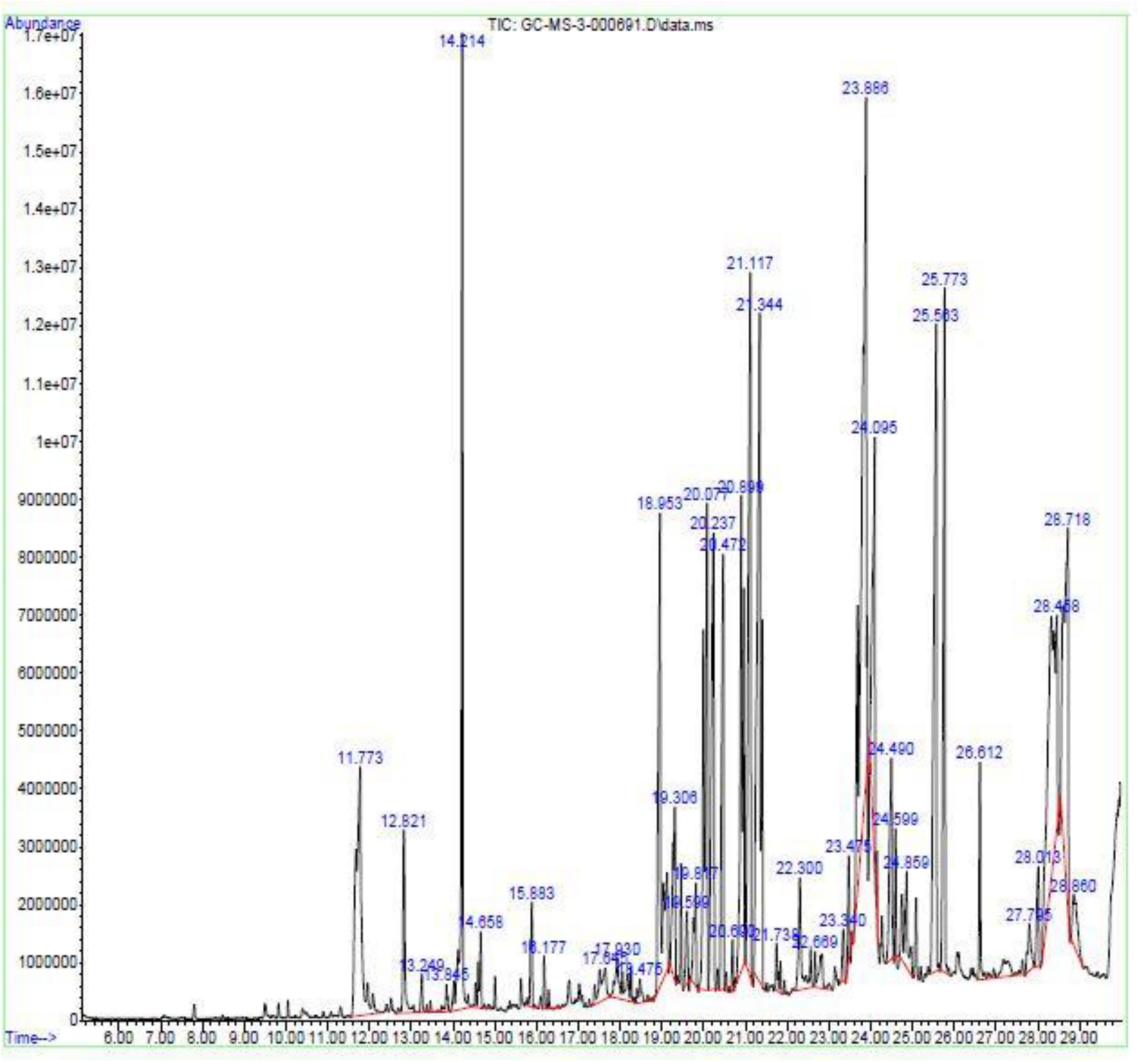
GC-MS Chromatogram of the ethyl acetate extract of *Streptomyces* sp. VITGV156.

**FIGURE 10:**
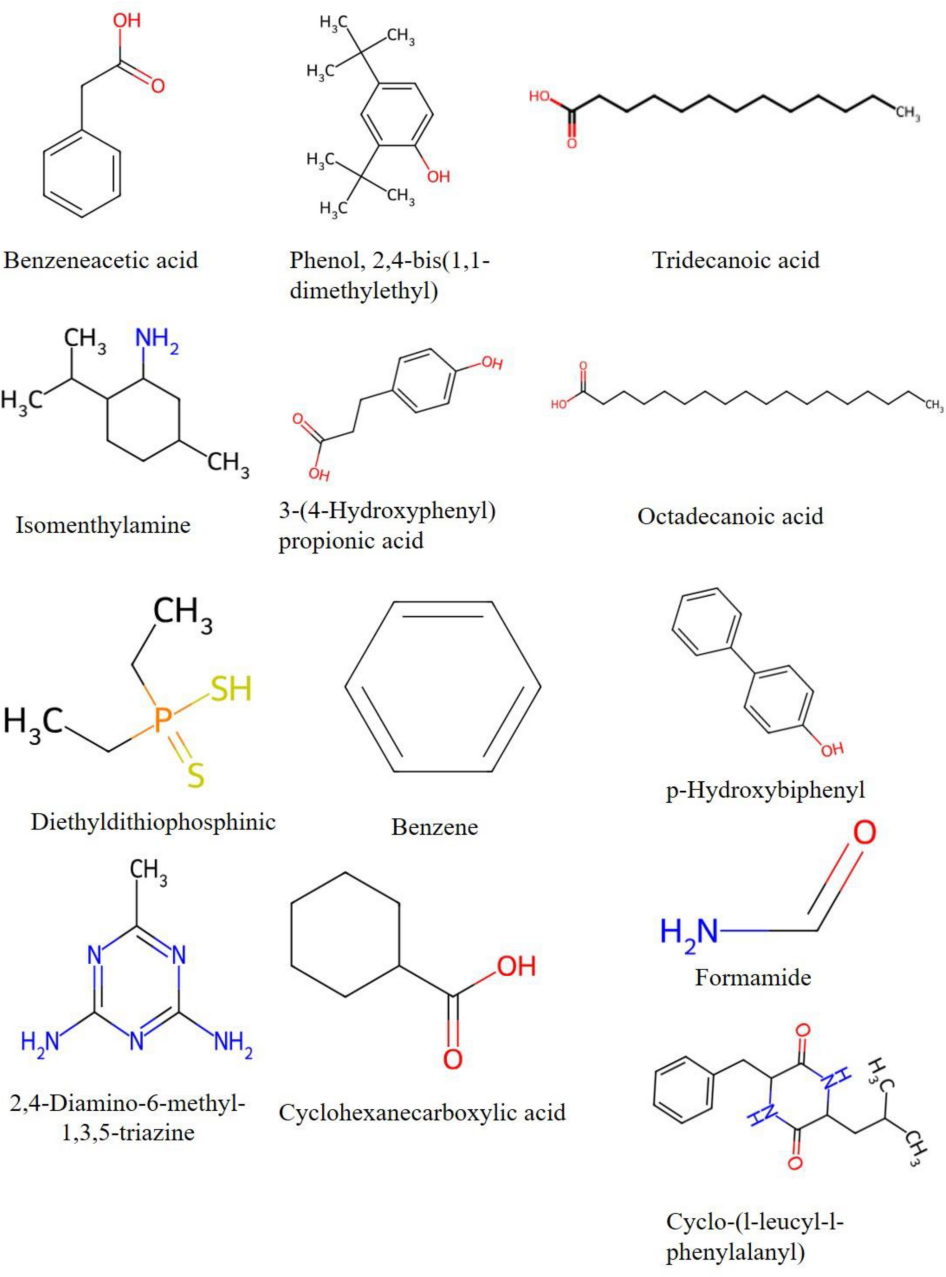
GC–MS chromatogram of *Streptomyces* sp. VITGV156 crude extract showing major metabolites identified by NIST library matching. Chemical structures of predominant compounds (relative peak area > 4%) are displayed adjacent to their corresponding chromatographic peaks. Identified compounds include benzeneacetic acid, phenol [2,4-bis(1,1-dimethylethyl)], tridecanoic acid, 3-(4-hydroxyphenyl) propionic acid, octadecanoic acid, p-hydroxybiphenyl, cyclo-(L-leucyl-L-phenylalanyl), and other significant constituents. Peak identification was based on spectral similarity index and retention time comparison with the NIST database.

GC–MS analysis was performed to experimentally characterize the chemical composition of the bioactive extract and to evaluate the presence of metabolites supporting genome-mining predictions. The total ion chromatogram (TIC) revealed a chemically diverse metabolite profile with 40 annotated compounds detected across the chromatographic run (Figure 9). Only peaks with NIST match factor ≥ 80% were retained (Supplementary Table 4). The detected metabolites belonged to multiple chemical classes: aromatic compounds, phenolic derivatives, fatty acids and long-chain hydrocarbons, nitrogen-containing heterocycles, diketopiperazines and organic acids and amide. Major abundant metabolites included: p-Hydroxybiphenyl (10.67%), Benzene (8.00%), Formamide (7.58%), Diethyldithiophosphinic acid (5.78%), Cyclo-(L-leucyl-L-phenylalanyl) (5.69%), 2,4-Diamino-6-methyl-1,3,5-triazine (5.77%), Phenol, 2,4-bis(1,1-dimethylethyl) (4.83%) and 3-(4-Hydroxyphenyl) propionic acid (4.31%). Importantly, several detected compounds—including phenolic derivatives, diketopiperazines, fatty acids, and aromatic metabolites—have previously been associated with antimicrobial activity. The observed metabolite diversity is consistent with the well-known secondary metabolite richness of *Streptomyces* species. Overall, GC–MS results confirm that *Streptomyces* sp. VITGV156 produces a chemically diverse pool of bioactive small molecules, providing experimental evidence supporting genome-mining predictions of extensive secondary metabolism. For improved visual interpretation, the chemical structures of the major metabolites identified through GC–MS analysis (relative peak area > 4%) are presented adjacent to their corresponding chromatographic peaks in Figure 10. Compound identification was performed based on spectral similarity matching using the NIST mass spectral library.

### LC–MS metabolomic evidence supporting genome-mined BGCs

**FIGURE 11:**
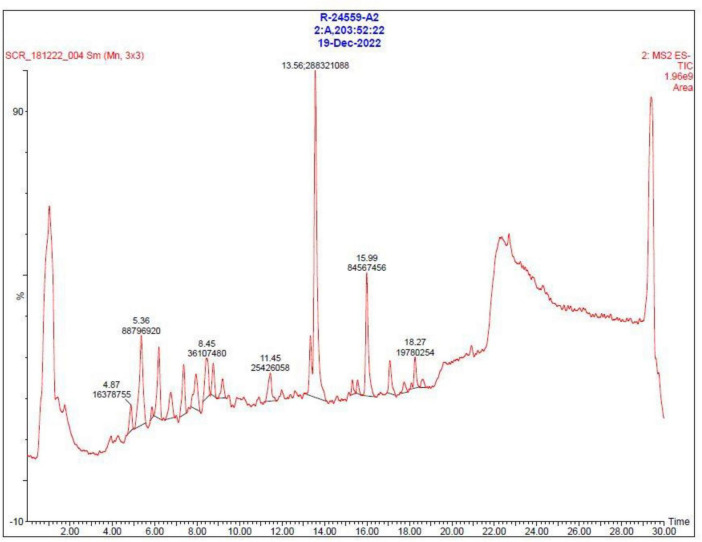
LC–MS base peak chromatogram (BPC) of the bioactive ethyl acetate extract of *Streptomyces* sp. VITGV156 analyzed in negative electrospray ionization (ESI–) mode using a QTOF mass spectrometer. The chromatogram shows multiple well-resolved peaks across the retention time range of 1–30 min, indicating the presence of chemically diverse secondary metabolites. The observed chromatographic complexity supports genome mining predictions of multiple biosynthetic gene clusters (BGCs), related metabolites.

**FIGURE 12:**
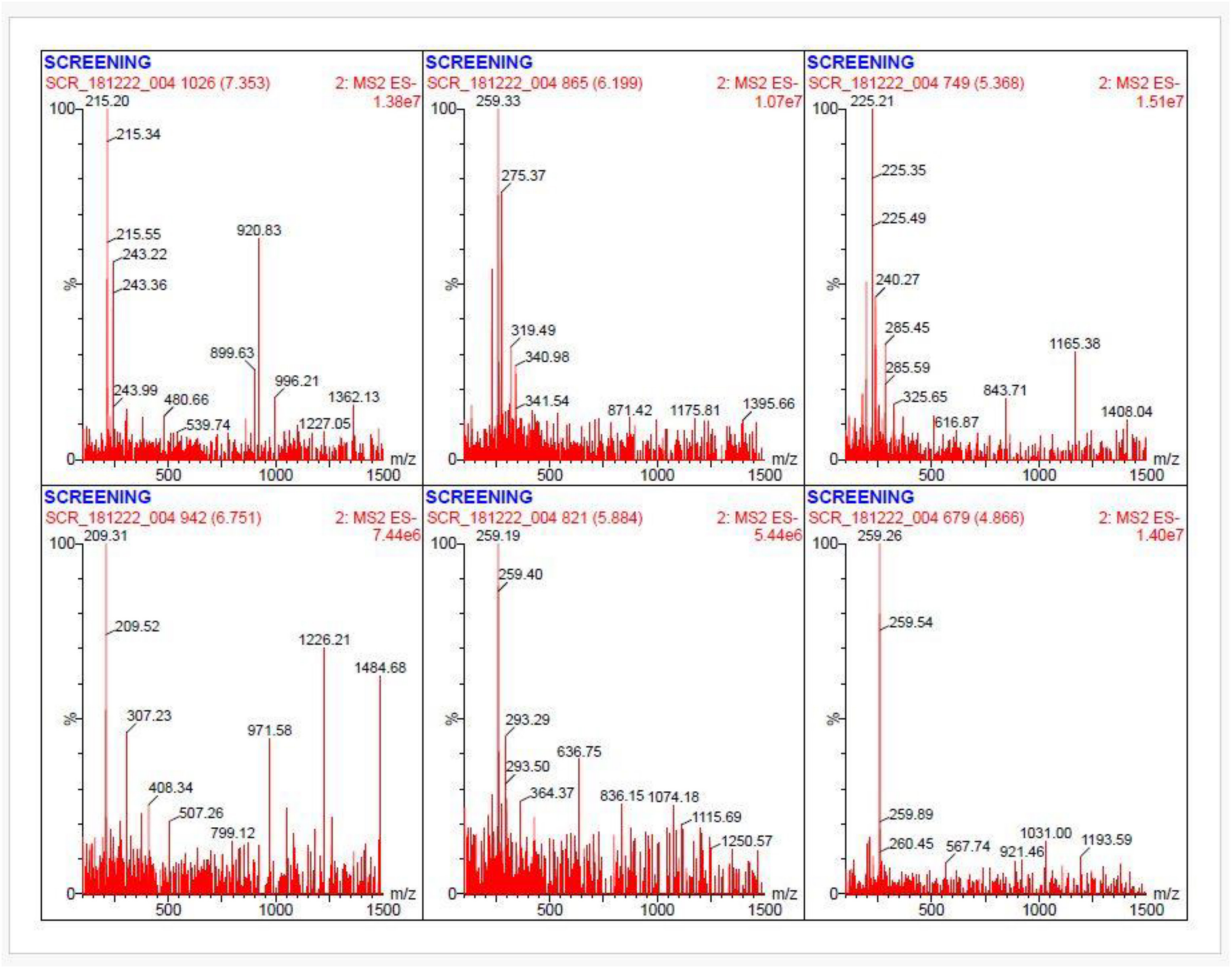
Representative LC–MS/MS fragmentation spectra of selected ions detected from the active ethyl acetate extract of *Streptomyces* sp. VITGV156 acquired in negative ESI mode. The spectra correspond to precursor ions eluting at different retention times, showing characteristic fragment ion patterns BGCS scaffolds. Several fragment profiles are consistent with metabolites biosynthesized by predicted antiSMASH BGCs, including terpene-associated clusters. These MS/MS data provide metabolomic support for genome-based predictions and highlight candidate metabolites.

To explore larger and non-volatile metabolites typically produced by biosynthetic gene clusters, LC–MS analysis of the bioactive extract was performed. LC–MS analysis of the crude ethyl acetate extract revealed multiple secondary metabolite peaks, and the annotated chromatogram and its mass spectrum were shown in Figures 11, 12, supporting the genome-based prediction of biosynthetic potential. The base peak chromatogram showed a complex metabolite profile with multiple well-resolved peaks across the chromatographic run. Prominent peaks were observed primarily between 12 and 27 min, indicating the presence of semi-polar and hydrophobic secondary metabolites characteristic of actinomycetes. High-resolution MS detected precursor ions spanning a wide range from m/z 209.31 to 1408.04, demonstrating substantial molecular diversity. The abundant ions detected in the m/z 350–1,000 region and several high-mass ions exceeded m/z 1,000. These mass ranges are consistent with polyketides, non-ribosomal peptides, and terpenoid metabolites predicted by antiSMASH analysis. Representative precursor ions detected m/z 215.34, 243.22 243.99, 209.31, 209.52, and 225.21. LC–MS/MS fragmentation of selected ions produced diagnostic fragment patterns characteristic of BGC-derived natural product scaffolds. Together, these complementary analyses bridge the gap between genome mining and chemical output, providing metabolomic evidence for the active expression of multiple biosynthetic gene clusters in *Streptomyces* sp. VITGV156.

Comparative Analysis of Genome-Mined and Experimentally Detected Metabolites

To integrate genome mining with metabolomic evidence, a comparative analysis was performed between antiSMASH-predicted biosynthetic products and compounds experimentally detected by GC–MS and LC–MS (Table 3). The comparison revealed three distinct categories: (i) metabolites predicted by genome mining but not detected chemically, (ii) compounds detected experimentally but not predicted by bioinformatics analysis, and (iii) putative overlaps supported by LC–MS mass proximity. Several predicted metabolites showed precursor ion masses consistent with LC–MS signals, suggesting active expression of specific biosynthetic gene clusters under the studied culture conditions. However, many genome-predicted compounds were not detected in the chemical analysis, indicating the likely presence of silent or cryptic biosynthetic gene clusters that may require specific environmental or regulatory triggers for activation. Conversely, GC–MS analysis identified numerous low-molecular-weight and volatile metabolites that were not predicted by antiSMASH. These compounds likely arise from primary metabolism, tailoring reactions, degradation products, or small-molecule pathways not captured by current genome-mining algorithms. This observation highlights the complementary nature of chemical and bioinformatic approaches and demonstrates that metabolomic diversity extends beyond predicted core BGC products. Importantly, LC–MS-based matches are considered putative and were assigned based on precursor ion mass proximity; definitive structural confirmation requires MS/MS spectral library matching and future structural validation studies. Overall, this integrative analysis strengthens the link between genomic potential and chemical expression while simultaneously emphasizing the existence of cryptic biosynthetic capacity within *Streptomyces* sp. VITGV156.

**TABLE 3:** Comparative analysis of metabolites predicted by genome mining and experimentally detected by GC–MS and LC–MS in *Streptomyces* sp. VITGV156.

Compound name	Molecular weight (Da)	Predicted by antiSMASH	Detected by GC–MS	Detected by LC–MS	Overlap status
Gaudimycin_A	340.3	Yes	No	Yes (m/z 340.98, 341.54)	Genome + LC–MS
Gaudimycin_C	356.3	Yes	No	*Yes (m/z 354.37)* *	Possible match
Ficellomycin	312.37	Yes	No	No	Genome only
Versipelostatin	1099.4	Yes	No	No clear 1,099 peak	Genome only
Herboxidiene	438	Yes	No	No	Genome only
Methylenomycin_A	182.17	Yes	No	No (closest 182 not observed)	Genome only
Naphthomycin_A	720.2	Yes	No	No	Genome only
Paulomycin	758.7	Yes	No	No	Genome only
Prejadomycin	324.3	Yes	No	Yes (m/z 325.65)	Genome + LC–MS
5-isoprenylindole-3-carboxylate	387.4	Yes	No	No	Genome only
Rabelomycin	338.3	Yes	No	No exact match	Genome only
Streptovaricin	769.8	Yes	No	No	Genome only
Lomofungin	314.25	Yes	No	No	Genome only
Beta-carotenoid	536.9	Yes	No	*Yes (m/z 539.74)* *	Possible match
Melanin	318.3	Yes	No	Yes (m/z 319.49)	Genome + LC–MS
Vicenistatin	500.7	Yes	No	*Yes (m/z 507.26)* *	Possible match
Isorenieratene	528.8	Yes	No	No	Genome only
Alpha-lipomycin	587.7	Yes	No	No	Genome only
Desferrioxamin_B	723.8	Yes	No	No	Genome only
Desferrioxamin_E	600.7	Yes	No	No	Genome only
Streptothricin	502.5	Yes	No	*Yes (m/z 507.26)* *	Possible match
Abaflavenone	218.33	Yes	No	*Yes (m/z 215.20–215.55)* *	Possible match
Coelibactin	400.4	Yes	No	No	Genome only
Coelichelin	565.6	Yes	No	No	Genome only
Ectoine	142.16	Yes	No	No	Genome only
Geosmin	182.3	Yes	No	No	Genome only
Germicidin	196.24	Yes	No	No	Genome only
Hopene_b	410.7	Yes	No	No	Genome only
Undecylprodigiosin	393.6	Yes	No	No	Genome only

LC–MS matches were assigned based on precursor ion mass proximity (± 2 Da tolerance) relative to predicted molecular weights; LC–MS identifications are considered putative and require further confirmation by MS/MS spectral library matching and structural validation; Compounds detected by GC–MS were annotated using the NIST library with a match factor ≥ 80%.; Genome-mined metabolites were predicted using antiSMASH analysis of the *Streptomyces* sp. VITGV156 genome.

“*” denotes compounds correlated with biosynthetic gene cluster predictions from antiSMASH and validated through LC–MS mass spectral profiling.

## Discussion

The present study combined experimental antibacterial screening with genome-guided computational prioritization to identify potential anti-infective metabolites from *Streptomyces* sp. VITGV156. The crude ethyl acetate extract demonstrated clear and concentration-dependent inhibition against both Gram-positive (*Bacillus subtilis* MTCC 2756; *Staphylococcus aureus* MTCC 737) and Gram-negative (*Escherichia coli* MTCC 1687; *Klebsiella pneumoniae* MTCC 109) pathogens. The absence of inhibition by DMSO confirmed that the activity was metabolite-driven rather than solvent-derived.

The observed inhibition zones (20–25 mm) indicate strong antibacterial activity for a crude extract, which is noteworthy because crude extracts typically contain mixed metabolites with varying polarity and bioactivity. Previous reports indicate that inhibition zones above 20 mm from crude *Streptomyces* extracts are generally considered indicative of potent antibacterial potential and justify further purification and characterization (Barka et al., 2015; Devine et al., 2017).

Importantly, the extract displayed comparable activity against Gram-negative bacteria, which are typically more resistant due to the presence of an outer membrane barrier. This suggests the presence of metabolites capable of penetrating or bypassing the lipopolysaccharide layer, a feature that is highly desirable for future antibiotic discovery (Silhavy et al., 2010). The slightly higher susceptibility of *K. pneumoniae* observed in this study is particularly relevant, given its classification as a critical priority pathogen by the World Health Organization (World Health Organization, 2021).

Although tetracycline exhibited larger inhibition zones, the VITGV156 extract achieved approximately 70–75% of tetracycline activity, which is significant considering that purified antibiotics are being compared with an unrefined metabolite mixture. Similar comparative trends have been reported in early-stage natural product discovery pipelines, where crude extracts often show partial but promising activity prior to compound isolation (Newman and Cragg, 2020).

The antibacterial activity observed experimentally is strongly supported by genomic evidence. *Streptomyces* species are widely recognized as the most prolific producers of antibiotics, contributing more than 70% of clinically used antibacterial agents (van der Meij et al., 2017). Genome sequencing has revealed that most *Streptomyces* strains contain 20–40 biosynthetic gene clusters (BGCs), many of which remain cryptic or silent under laboratory conditions (Rutledge and Challis, 2015).

Recent advances in genome mining have fundamentally transformed the discovery of secondary metabolites from *Streptomyces*. AntiSMASH-based analyses, comparative genomics, and metabolomics-guided prioritization have been widely used to identify cryptic biosynthetic gene clusters and link them with chemical products (Medema and Fischbach, 2015). For example, genome-guided approaches have enabled the discovery of previously uncharacterized polyketides, non-ribosomal peptides, and hybrid metabolites from diverse *Streptomyces* species (Ziemert et al., 2016). Integrative strategies combining genomics with LC–MS/MS-based molecular networking and metabolomics have further improved the ability to connect predicted biosynthetic pathways with expressed metabolites, reducing the rediscovery of known compounds and accelerating antibiotic discovery pipelines (Duncan et al., 2015). These studies highlight the growing importance of combining genome mining with experimental validation in modern natural product research.

The integration of genome mining with experimental validation has therefore become a powerful strategy to accelerate antibiotic discovery. In this context, the broad-spectrum antibacterial activity of VITGV156 provides experimental confirmation that at least a subset of its predicted BGCs are functionally expressed and produce bioactive metabolites.

A major strength of this study is the selection of clinically relevant resistance proteins as docking targets. Tet(X4) is a recently emerged flavin-dependent monooxygenase capable of inactivating tigecycline, a last-resort antibiotic used against multidrug-resistant pathogens (Cheng et al., 2021). Similarly, QnrB1 protects bacterial DNA gyrase from fluoroquinolone inhibition and contributes to plasmid-mediated quinolone resistance (Briales et al., 2010).

Targeting resistance proteins rather than traditional essential enzymes represents an emerging strategy in antibiotic development. Inhibitors of resistance enzymes can restore the activity of existing antibiotics, thereby extending the lifespan of current drugs and reducing the emergence of resistance (Brown and Wright, 2016). The docking strategy used in this study is therefore aligned with contemporary approaches in antimicrobial research.

The docking validation step yielded an RMSD value of 1.71 Å, confirming the reliability of the docking protocol. RMSD values below 2 Å are widely accepted as evidence of accurate docking reproduction of crystallographic ligand poses (Hevener et al., 2009). The virtual screening of 29 predicted secondary metabolites revealed multiple compounds with strong binding affinities toward both resistance proteins. Binding energies up to -12.3 kcal/mol suggest stable protein–ligand interactions comparable to known inhibitors. Among the screened metabolites, vicenistatin, prejadomycin, and ectoine emerged as the most promising candidates based on combined docking, PASS prediction, and toxicity screening.

Vicenistatin is an aminoglycoside-like macrolactam antibiotic previously reported to exhibit antitumor and antimicrobial activity (Ogasawara et al., 2004). Its strong docking performance and favorable toxicity profile suggest an additional potential role in resistance inhibition. Prejadomycin belongs to the angucycline family, a class of aromatic polyketides known for diverse antibacterial and anticancer activities (Kharel et al., 2004). Ectoine, although primarily known as an osmoprotectant, has recently been associated with stress-protection and biofilm modulation, which may contribute indirectly to antimicrobial effects (Pastor et al., 2010). The convergence of bioactivity prediction, docking, and toxicity filtering strengthens the prioritization process and reduces the likelihood of false positives, a common limitation in purely computational drug discovery workflows.

Molecular dynamics simulations provided further evidence supporting vicenistatin as the top candidate. The reduction in RMSD, RMSF, SASA, and radius of gyration upon ligand binding indicates enhanced structural stability of both Tet(X4) and QnrB1 complexes. Persistent hydrogen bonding throughout the simulation further confirms stable ligand retention within the binding pockets. MD simulations are increasingly used to validate docking predictions because they capture protein flexibility and solvent effects that static docking cannot account for (Hollingsworth and Dror, 2018). The improved compactness and reduced flexibility observed in the ligand-bound complexes suggest that vicenistatin may effectively stabilize inactive conformations of resistance proteins.

The integration of experimental antibacterial screening with genome-guided computational prioritization represents a key strength of this study (Farha et al., 2025). Traditional antibiotic discovery often suffers from high rediscovery rates and low success in translating *in vitro* activity into viable drug candidates (Muteeb et al., 2023; Blaskovich and Cooper, 2025). By combining wet-lab validation with *in silico* prioritization and toxicity filtering, the present workflow helps address this challenge and provides a rational strategy for accelerating antibiotic discovery from *Streptomyces*.

Despite the promising findings, several limitations must be acknowledged. The antibacterial activity was evaluated using crude extracts, and the specific active compounds remain to be isolated and experimentally validated. Additionally, docking and MD simulations provide predictive evidence but require biochemical and microbiological validation to confirm resistance inhibition.

Future work should focus on metabolite purification, MIC determination, synergy studies with existing antibiotics, and *in vitro* enzyme inhibition assays. Such studies will be essential to validate vicenistatin and related metabolites as potential resistance-modifying agents. Overall, the results demonstrate that *Streptomyces* sp. VITGV156 represents a promising source of antibacterial metabolites with potential activity against antibiotic resistance mechanisms. The combined experimental and computational workflow provides a robust framework for prioritizing candidate molecules and supports further investigation toward antibiotic discovery.

Despite these promising findings, several limitations must be acknowledged. The antibacterial activity was evaluated using crude extracts, and the specific active compounds remain to be isolated and experimentally validated. In addition, genome mining, docking, and molecular dynamics simulations provide predictive evidence rather than direct proof of biological activity. Future work will therefore focus on involving pathway activation strategies, heterologous expression, and gene knockout experiments will be necessary to establish definitive genotype–phenotype relationships, metabolite purification, Minimal inhibitory determination, resistance-enzyme inhibition assays, and synergy studies with existing antibiotics to validate the prioritized metabolites as resistance-modifying agents.

## Conclusion

This study integrates antibacterial screening with genome mining and structure-based computational prioritization to identify resistance-targeting metabolites from *Streptomyces* sp. VITGV156. The crude extract exhibited broad-spectrum antibacterial activity against both Gram-positive and Gram-negative pathogens, supporting the strain’s bioactive potential. Genome-guided virtual screening identified multiple candidate metabolites with strong binding affinity toward the resistance proteins QnrB1 and Tet(X4), with vicenistatin emerging as the most promising candidate based on docking, bioactivity prediction, toxicity filtering, and molecular dynamics simulations. By combining experimental evidence with computational prioritization, this work provides a practical framework for accelerating the discovery of resistance-modifying natural products and establishes a foundation for future purification and biochemical validation of prioritized metabolites.

## Data Availability

The datasets presented in this study can be found in online repositories. The names of the repository/repositories and accession number(s) can be found at: https://www.ncbi.nlm.nih.gov/, SRS9645416.

## Footnotes

1
https://www.ncbi.nlm.nih.gov/bioproject/PRJNA750872

2
https://www.rcsb.org/
