# Supplementary material for: Genome-guided discovery and computational prioritization of next generation drug development from Streptomyces sp. VITGV156 (MCC 4965)
Source: Front Microbiol. 2026 Mar 30;17:1736442. doi: 10.3389/fmicb.2026.1736442 (PMC13070913; doi:10.3389/fmicb.2026.1736442)
Supplement: Supplementary file 1 [file Data_Sheet_1.docx]

**Supplementary File**

**Genome-Guided and In Silico Prioritization of Antibacterial Secondary Metabolites from Streptomyces sp. VITGV156 (MCC 4965)**

## Table 1. Molecular docking results showing binding affinity (Gibbs free energy, kcal/mol) of predicted secondary metabolites against target proteins 2XTY and 7EPV.

| **S. No.** | **Secondary Metabolites** | **2XTY**  **(kcal/mol)** | **7EPV**  **(kcal/mol)** |
| --- | --- | --- | --- |
| 1 | Gaudimycin_A | -8.1 | -12.2 |
| 2 | Gaudimycin_C | -7 | -10.2 |
| 3 | Ficellomycin | -5.4 | -7.5 |
| 4 | Versipelostatin | -8.1 | -8.7 |
| 5 | Herboxidiene | -6.1 | -7.7 |
| 6 | Methylenomycin_A | -4.4 | -6.1 |
| 7 | Naphthomycin_A | -9.4 | -11.3 |
| 8 | Paulomycin | -6.6 | -6.4 |
| 9 | Prejadomycin | -8.9 | -9.3 |
| 10 | 5-isoprenylindole-3-carboxylate beta-D-glycosyl ester | -7.6 | -9.9 |
| 11 | Rabelomycin | -7.3 | -9.2 |
| 12 | Streptovaricin | -7.1 | -9.1 |
| 13 | Lomofungin | -6.3 | -8.5 |
| 14 | Beta-Carotenoid | -7 | -8 |
| 15 | Melanin | -7.4 | -9.6 |
| 16 | Vicenistatin | -9.5 | -12.3 |
| 17 | Isorenieratene | -7.2 | -9.4 |
| 18 | Alpha-lipomycin | -8 | -9.8 |
| 19 | Desferrioxamin_B | -6.5 | -8.3 |
| 20 | Desferrioxxamin_E | -8.9 | -11.4 |
| 21 | Streptothricin | -6.9 | -8.6 |
| 22 | Abaflavenone | -5.1 | -7.9 |
| 23 | Coelibactin | -7.5 | -11.3 |
| 24 | Coelichelin | -6.8 | -8 |
| 25 | Ectoine | -7.2 | -9.4 |
| 26 | Geosmin | -4.5 | -6.2 |
| 27 | Germicidin | -4.6 | -6.1 |
| 28 | Hopene_b | -7.1 | -9.2 |
| 29 | Undecylprodigiosin | -5.4 | -7.1 |

Footnote: Binding affinities were calculated using AutoDock Vina. More negative ΔG values indicate stronger predicted binding interactions. Proteins 2XTY and 7EPV were retrieved from the Protein Data Bank (PDB).

**Table 2**. **PASS-predicted biological activity profiles of antiSMASH-identified secondary metabolites from Streptomyces sp. VITGV156.**

| S. No. | Secondary Metabolites | Similarity score | Antibacterial  Activity  (Pa-value) | Antifungal activity  (Pa-value) | Antibiotic activity  (Pa-value) |
| --- | --- | --- | --- | --- | --- |
| 1 | Gaudimycin_A | 1 | 0.362 | 0.506 | 0.204 |
| 2 | Gaudimycin_C | 1 | 0.445 | 0.607 | 0.306 |
| 3 | Ficellomycin | 3 | 0.342 | - | 0.408 |
| 4 | Versipelostatin | 5 | 0.878 | 0.668 | 0.799 |
| 5 | Herboxidiene | 8 | 0.558 | 0.832 | 0.356 |
| 6 | Methylenomycin_A | 9 | 0.406 | 0.341 | 0.254 |
| 7 | Naphthomycin A | 9 | 0.683 | 0.747 | 0.446 |
| 8 | Paulomycin | 9 | 0.263 | 0.245 | 0.378 |
| 9 | **Prejadomycin** | **9** | **0.865** | **0.845** | **0.796** |
| 10 | 5-isoprenylindole-3-carboxylate beta-D-glycosyl ester | 23 | 0.517 | 0.276 | 0.231 |
| 11 | Rabelomycin | 27 | 0.485 | 0.547 | 0.207 |
| 12 | Streptovaricin_U | 29 | 0.586 | 0.617 | 0.342 |
| 13 | Lomofungin | 34 | 0.329 | 0.305 | 0.156 |
| 14 | Beta-Carotene | 45 | 0.293 | 0.383 | 0.161 |
| 15 | Melanin | 60 | 0.164 | 0.049 | 0.107 |
| 16 | **Vicenistatin** | **60** | **0.781** | **0.781** | **0.634** |
| 17 | Isorenieratene | 63 | 0.283 | 0.402 | 0.16 |
| 18 | Alpha-lipomycin | 72 | 0.753 | 0.75 | 0.622 |
| 19 | Desferrioxamin_B | 83 | 0.326 | 0.197 | 0.233 |
| 20 | Desferrioxxamin_E | 83 | 0.342 | 0.265 | 0.186 |
| 21 | Streptothricin | 95 | 0.665 | - | 0.266 |
| 22 | Abaflavenone | 100 | 0.266 | 0.233 | 0.145 |
| 23 | Coelibactin | 100 | - | - | - |
| 24 | Coelichelin | 100 | 0.512 | 0.277 | 0.186 |
| 25 | **Ectoine** | **100** | **0.654** | **0.768** | **0.831** |
| 26 | Geosmin | 100 | 0.203 | 0.281 | 0.107 |
| 27 | Germicidin | 100 | 0.495 | 0.505 | 0.286 |
| 28 | Hopene_b | 100 | 0.295 | 0.434 | 0.093 |
| 29 | Undecylprodigiosin | 100 | - | - | - |

Footnote: Biological activity prediction was performed using the PASS online server. Pa (probability of activity) values range from 0 to 1, where higher values indicate greater likelihood of biological activity. “–” indicates values below the prediction threshold.

**Table S3. Two-way ANOVA results for antibacterial activity of the crude extract and tetracycline.**

| **Organism** | **Source of variation** | **F (DFn, DFd)** | **p-value** | **Significance** |
| --- | --- | --- | --- | --- |
| ***Staphylococcus aureus*** | Interaction | F (6,24) =14.41 | <0.0001 | **** |
|  | Treatment (row factor) | F (3,24) =57.23 | <0.0001 | **** |
|  | Volume (column factor) | F (2,24) =13242 | <0.0001 | **** |
| ***Bacillus subtilis*** | Interaction | F (6,24) =4.70 | 0.0027 | ** |
|  | Treatment | F (3,24) =9.41 | 0.0003 | *** |
|  | Volume | F (2,24) =5760 | <0.0001 | **** |
| ***Klebsiella pneumoniae*** | Interaction | F (6,24) =2.10 | 0.0907 | ns |
|  | Treatment | F (3,24) =8.10 | 0.0007 | *** |
|  | Volume | F (2,24) =1446 | <0.0001 | **** |
| ***Escherichia coli*** | Interaction | F (6,24) =4.70 | 0.0027 | ** |
|  | Treatment | F (3,24) =10.96 | 0.0001 | *** |
|  | Volume | F (2,24) =8933 | <0.0001 | **** |

**Significance:** ns = not significant; *p < 0.05; **p < 0.01; ***p < 0.001; ****p < 0.0001.

**Table S4**. Antimicrobial compounds predicted from the GC-MS with NIST database for the crude extract of *Streptomyces* sp. VITG156

| **S. No** | **Chemical Compound** | **RT** | **Molecular weight** | **Molecular Formula** | **Area %** |
| --- | --- | --- | --- | --- | --- |
| **1** | **Benzeneacetic acid** | **11.773** | **136.15** | **C_8_H_8_NO_2_** | **4.83** |
| 2 | Indole | 12.821 | 117.15 | C_8_H_7_N | 1.25 |
| 3 | Cyclodecane | 13.249 | 140.27 | C_10_H_20_ | 0.27 |
| 4 | Benzamide | 13.845 | 121.14 | C_7_H_7_NO | 0.20 |
| **5** | **Phenol, 2,4-bis(1,1-dimethylethyl)-** | **14.214** | **206.32** | **C_4_H_22_O** | **4.83** |
| 6 | Benzeneacetamide | 14.658 | 135.16 | C_8_H_9_NO | 0.63 |
| 7 | Acetamide, N-(2-phenylethyl)- | 15.883 | 2555.2 | C_134_H_132_N_34_O_7_S_7_ | 0.63 |
| 8 | 1-Nonadecene | 16.177 | 266.5 | C_19_H_38_ | 0.28 |
| 9 | 1,3-Cyclopentanedione | 17.645 | 98.1 | C_5_H_6_O_2_ | 0.77 |
| 10 | Benzoic acid | 17.930 | 122.12 | C_7_H_6_O_2_ | 0.98 |
| 11 | n-Hexadecanoic acid | 18.475 | 400.8 | C_22_H_48_O_2_Si_2_ | 0.29 |
| **12** | **Tridecanoic acid** | **18.953** | **280.4** | **C_18_H_32_O_2_** | **4.69** |
| 13 | 3-Pyrrolidin-2-yl-propionic acid | 19.306 | 219.28 | C_13_H_17_NO_2_ | 1.54 |
| 14 | Piperidin-4-one | 19.599 | 99.13 | C_5_H_9_NO | 0.44 |
| 15 | Cyclo-(glycyl-l-leucyl) | 19.817 | 170.21 | C_8_H_14_N_2_O_2_ | 0.86 |
| **16** | **Isomenthylamine** | **20.077** | **155.285** | **C_10_H_21_N** | **4.58** |
| 17 | **3-(4-Hydroxyphenyl) propionic acid** | **20.237** | **166.17** | **C_9_H_10_O_3_** | **4.31** |
| 18 | Phenol, 3,5-dimethoxy- | 20.472 | 2630.8 | C_150_H_203_ClFN_27_O_12_ | 2.93 |
| 19 | 2,5-Piperazinedione | 20.690 | 114.1 | C_4_H_6_N_2_ | 0.19 |
| 20 | **Octadecanoic acid** | **20.899** | **883.0** | **C_18_H_36_O_2_** | **4.21** |
| 21 | **Diethyldithiophosphinic acid** | **21.117** | **154.2** | **C_4_H_11_PS_2_** | **5.78** |
| 22 | **Benzene** | **21.344** | **78.11** | **C_6_H_6_** | **8.00** |
| 23 | L-Proline | 21.738 | 115.13 | C_5_H_9_NO_2_ | 0.35 |
| 24 | Benzoic acid | 22.300 | 122.12 | C_7_H_6_O_2_ | 1.18 |
| 25 | Nonadecane | 22.669 | 268.5 | C_19_H_40_ | 0.59 |
| 26 | Naphthalene | 23.340 | 128.169 | C_10_H_8_ | 0.34 |
| 27 | 2,5-Piperazinedione | 23.475 | 114.1 | C_4_H_6_N_2_O_2_ | 0.50 |
| 28 | **p-Hydroxybiphenyl** | **23.886** | **170.21** | **C_12_H_10_O** | **10.67** |
| 29 | 2,5-Piperazinedione | 24.095 | 114.1 | C_4_H_6_N_2_ | 3.48 |
| 30 | 2,5-Piperazinedione | 24.095 | 114.1 | C_4_H_6_N_2_ | 3.48 |
| 31 | L-Proline | 24.599 | 115.13 | C_5_H_9_NO_2_ | 0.37 |
| 32 | 3-Methyl-5-phenylpyridine | 24.859 | 169.22 | C_12_H_11_N | 1.27 |
| 33 | **2,4-Diamino-6-methyl-1,3,5-triazine** | **25.563** | **125.13** | **C_4_H_7_N_5_** | **5.77** |
| 34 | **Cyclohexanecarboxylic acid** | **25.773** | **128.17** | **C_7_H_12_O_2_** | **4.36** |
| 35 | 13-Docosenamide, (Z)- | 26.612 | 337.6 | C_22_H_43_NO | 1.62 |
| 36 | Phenol | 27.795 | 94.11 | C_6_H_6_O | 0.51 |
| 37 | Aminopyrazine | 28.013 | 95.1 | C_4_H_5_N_3_ | 0.74 |
| 38 | **Formamide** | **28.458** | **45.041** | **CH_3_NO** | **7.58** |
| 39 | **Cyclo-(l-leucyl-l-phenylalanyl)** | **28.718** | **260.329** | **C_15_H_20_N_2_O_2_** | **5.69** |
| 40 | Glycyl-L-tyrosine | 28.860 | 238.24 | C_11_H_14_N_2_O_4_ | 0.87 |

RT = retention time (min); Area % = relative peak area percentage.


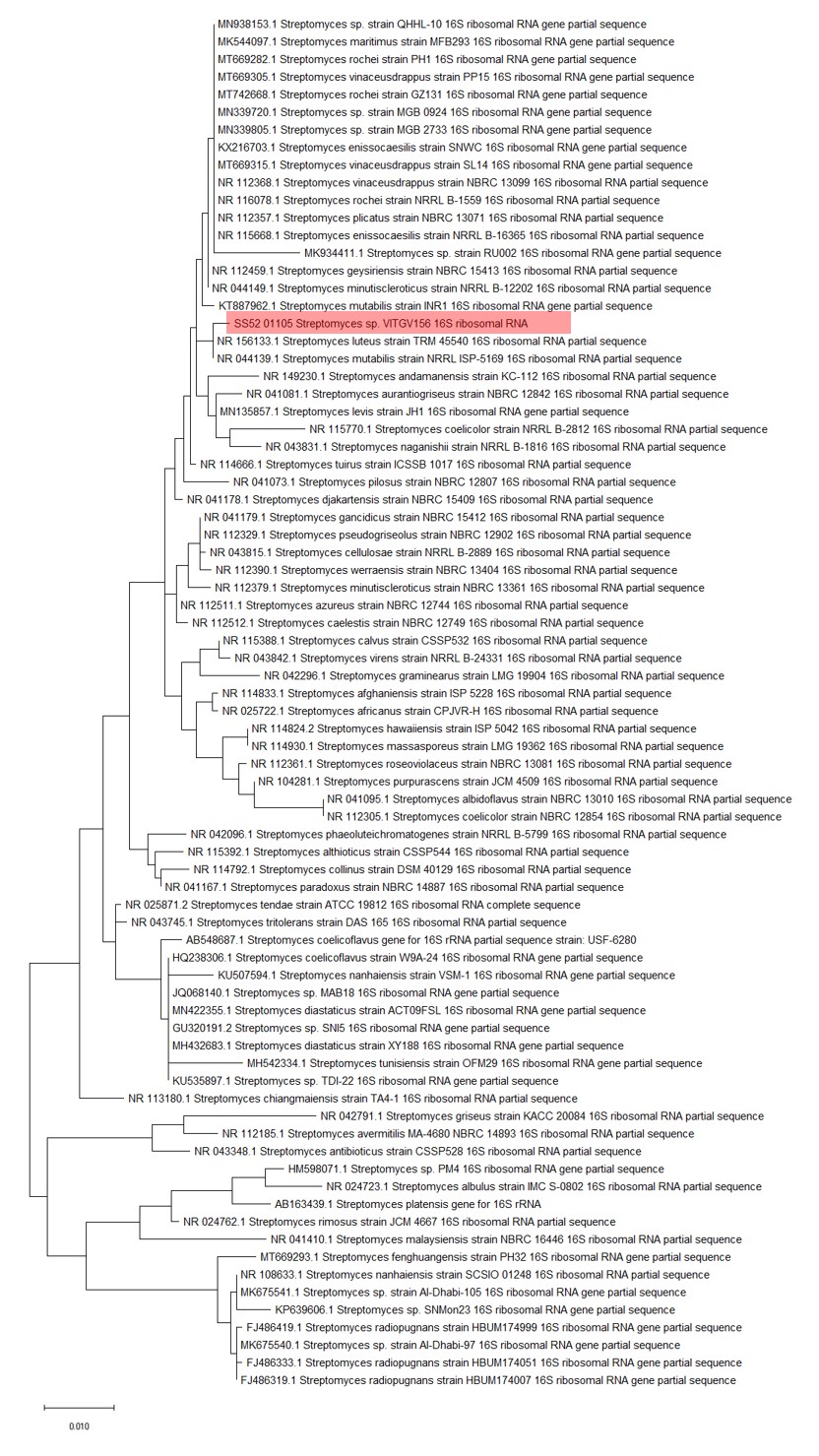


**Figure S1.** Phylogenetic tree based on 16S rRNA gene sequences showing the taxonomic position of Streptomyces sp. VITGV156. The tree was constructed using the Maximum Likelihood method with 1,000 bootstrap replicates in MEGA X. Bootstrap values (>50%) are shown at branch nodes. Accession numbers are indicated after species names. Type species are marked with (T). The studied strain is highlighted in bold.


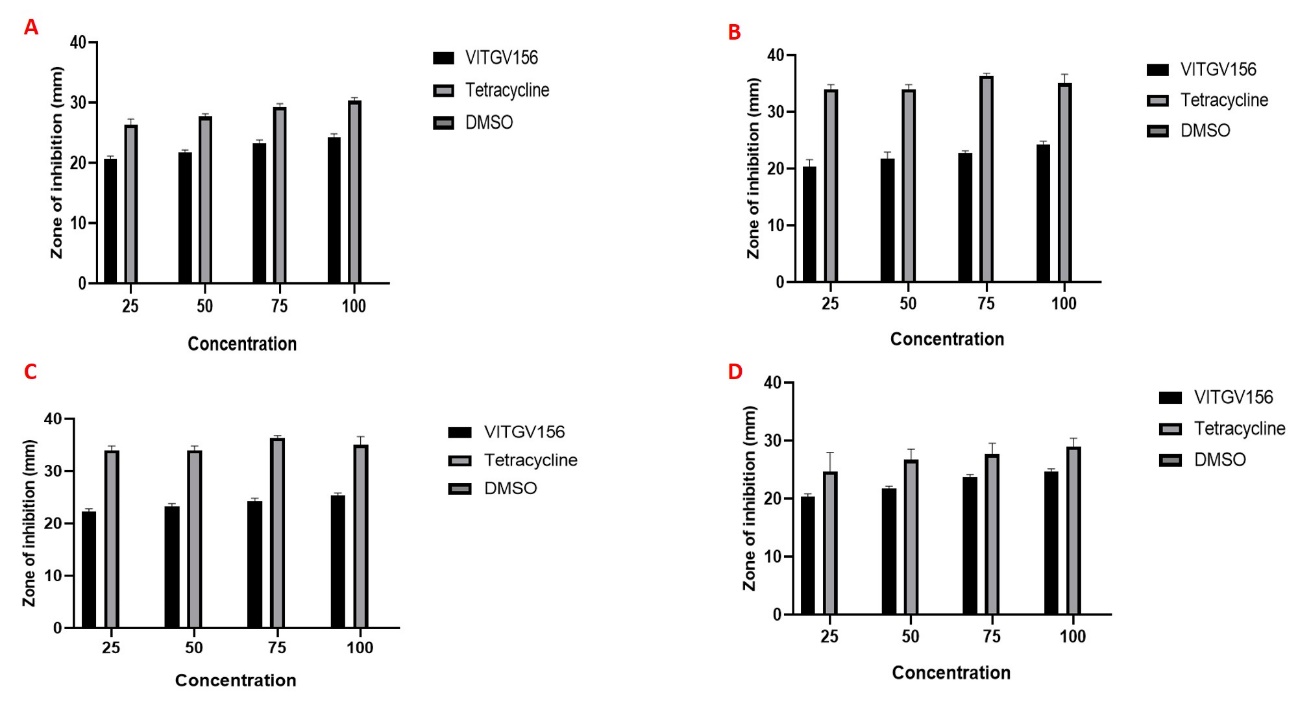


### ****Figure S2. Statistical analysis of antibacterial activity using two-way ANOVA.****

Two-way ANOVA was performed to evaluate the effects of treatment (crude extract vs tetracycline) and volume (25–100 µL) on inhibition zone diameters for each tested organism. (A) Staphylococcus aureus, (B) Bacillus subtilis, (C) Klebsiella pneumoniae, and (D) Escherichia coli. For all organisms, the treatment factor and volume factor showed statistically significant effects (p < 0.05). Interaction between treatment and volume was significant for S. aureus, B. subtilis, and E. coli, while a non-significant interaction was observed for K. pneumoniae. Detailed ANOVA outputs including F-values, degrees of freedom, and p-values are provided in Table S1.
